# A computational map of the human-SARS-CoV-2 protein–RNA interactome predicted at single-nucleotide resolution

**DOI:** 10.1093/nargab/lqad010

**Published:** 2023-02-20

**Authors:** Marc Horlacher, Svitlana Oleshko, Yue Hu, Mahsa Ghanbari, Giulia Cantini, Patrick Schinke, Ernesto Elorduy Vergara, Florian Bittner, Nikola S Mueller, Uwe Ohler, Lambert Moyon, Annalisa Marsico

**Affiliations:** Computational Health Center, Helmholtz Center Munich, Munich, Germany; Computational Health Center, Helmholtz Center Munich, Munich, Germany; Computational Health Center, Helmholtz Center Munich, Munich, Germany; Informatics 12 Chair of Bioinformatics, Technical University Munich, Garching, Germany; Institutes of Biology and Computer Science, Humboldt University, Berlin, Germany; Max Delbruck Center, Computational Regulatory Genomics, Berlin, Germany; Computational Health Center, Helmholtz Center Munich, Munich, Germany; Computational Health Center, Helmholtz Center Munich, Munich, Germany; Computational Health Center, Helmholtz Center Munich, Munich, Germany; Knowing01 GmbH, Munich, Germany; Knowing01 GmbH, Munich, Germany; Institutes of Biology and Computer Science, Humboldt University, Berlin, Germany; Max Delbruck Center, Computational Regulatory Genomics, Berlin, Germany; Computational Health Center, Helmholtz Center Munich, Munich, Germany; Computational Health Center, Helmholtz Center Munich, Munich, Germany

## Abstract

RNA-binding proteins (RBPs) are critical host factors for viral infection, however, large scale experimental investigation of the binding landscape of human RBPs to viral RNAs is costly and further complicated due to sequence variation between viral strains. To fill this gap, we investigated the role of RBPs in the context of SARS-CoV-2 by constructing the first *in silico* map of human RBP-viral RNA interactions at nucleotide-resolution using two deep learning methods (pysster and DeepRiPe) trained on data from CLIP-seq experiments on more than 100 human RBPs. We evaluated conservation of RBP binding between six other human pathogenic coronaviruses and identified sites of conserved and differential binding in the UTRs of SARS-CoV-1, SARS-CoV-2 and MERS. We scored the impact of mutations from 11 variants of concern on protein–RNA interaction, identifying a set of gain- and loss-of-binding events, as well as predicted the regulatory impact of putative future mutations. Lastly, we linked RBPs to functional, OMICs and COVID-19 patient data from other studies, and identified MBNL1, FTO and FXR2 RBPs as potential clinical biomarkers. Our results contribute towards a deeper understanding of how viruses hijack host cellular pathways and open new avenues for therapeutic intervention.

## INTRODUCTION

SARS-CoV-2, causative agent of the recent COVID-19 pandemic, has and still is affecting the lives of billions of people worldwide. Despite the large-scale vaccination effort, the number of infections and deaths remains high, primarily among the non-vaccinated and otherwise vulnerable individuals. Difficulty to control SARS-CoV-2 infections is partly due to the continuous emergence of novel viral variants, against which the full efficacy of current vaccines is still debated, as well as the lack of effective medication. This calls for a better understanding of the biology of SARS-CoV-2 to design alternative therapeutic strategies. SARS-CoV-2 is a betacoronavirus with a positive-sense, single-stranded RNA of 30kb (1). Upon infection, the released RNA molecule depends on the host cell’s protein synthesis machinery to express a set of viral proteins crucial for replication (2). The genomic RNA is translated to produce non-structural proteins (nsps) from two open reading frames (ORFs), ORF1a and ORF1b, and it also contains untranslated regions (UTRs) at the 5′ and 3′ ends of the genomic RNA (1). A recent study revealed the complexity of the SARS-CoV-2 transcriptome, due to numerous discontinuous transcription events (3). Negative sense RNA intermediates are generated to serve as the template for the synthesis of positive-sense genomic RNA (gRNA) and subgenomic RNAs (sgRNA) which encode conserved structural proteins (spike protein [S], envelop protein [E], membrane protein [M] and nucleocapsid protein [N]), and several accessory proteins (3a, 6, 7a, 7b, 8 and 10) (3). During its life cycle, SARS-CoV-2 extensively interacts with host factors in order to facilitate cell entry, transcription of viral RNA and translation of subgenomic mRNAs, virion maturation and evasion of the host’s immune response (1,4,5). Mechanisms of virus-host interaction are multifaceted and include protein–protein interactions (PPIs), binding of viral proteins to the host transcriptome (6), RNA–RNA interactions and binding of host proteins to viral RNAs. Studies on SARS-CoV-2 infected cells to date have predominantly focused on the entry of SARS-CoV-2 into human epithelial cells, which involves the interaction of the viral spike protein S with the human ACE2 receptor (3). Other studies characterized changes in the host cell transcriptome and proteome upon infection and identified host factors essential for viral replication via CRISPR screenings (7–9).

Lastly, mapping of protein–protein interactions (PPIs) between viral and host proteins has revealed cellular pathways important for SARS-CoV-2 infection. For instance, a recent study identified close to 300 host-virus interactions in the context of SARS-CoV-2 (8).

However, these studies have been of limited impact with respect to revealing how the viral RNA is regulated during infection.

RNA viruses hijack key cellular host pathways by interfering with the activity of master regulatory proteins, including RNA binding proteins (RBPs) (10). RBPs are a family of proteins that bind to RNA molecules and control several aspects of cellular RNA metabolism, including splicing, stability, export and translation initiation. Often, RNA targets of an RBP share at least one common local sequence or structural feature which facilitates the recognition of the RNA by the protein. Host cell RBPs have previously been reported to interact with viral RNA elements and influence several steps of the viral life cycle, such as recruitment of viral RNA to the membrane and synthesis of subgenomic viral RNAs (11,12,13). Indeed, in a recent proteome-wide study, 342 RBPs were identified to be annotated with gene ontology (GO) terms related to viruses, infection or immunity with a further 130 RBPs being linked to viruses in literature (13). Examples include the Dengue virus (14), the Murine Norovirus (MNV) (15) and Sindbis virus (SINV), where it has been shown that RBPs stimulated by the infection redistribute to viral replication factories and modulate the success of infection (13). The ability of viral RNAs to recruit essential host RBPs could explain permissiveness of certain cell types as well as its range of hosts (11), which is especially relevant for zoonotic viruses such as SARS-CoV-2. In the context of SARS-CoV infection, DEAD-box helicase 1 (DDX1) RBP has been shown to facilitate template read-through and thus replication of genomic viral RNA, while heterogeneous nuclear ribonucleoprotein A1 (hnRNPA1) might regulate viral RNA synthesis (5,16,17). Multiple recent studies show that SARS-CoV-2 RNAs extensively interact with both pro-and anti-viral host RBPs during its life cycle (18–21). Using comprehensive identification of RNA-binding proteins by mass spectrometry (ChIRP-MS), Flynn *et al.* (18) identified a total of 229 vRNA-bound host factors in human Huh7.5 cells with prominent roles in protecting the host from virus-induced cell death. Schmidt *et al.* (19) identified 104 vRNA-bound human proteins in the same cell line via RNA antisense purification and quantitative mass spectrometry (RAP-MS), with GO-terms strongly enriched in translation initiation, nonsense-mediated decay and viral transcription. The authors further confirmed the specific location of vRNA binding sites for cellular nucleic acid-binding protein (CNBP) and La-related protein 1 (LARP1) via enhanced cross-linking immunoprecipitation followed by sequencing (eCLIP-seq), which were both associated to restriction of SARS-CoV-2 replication (19). Lee at al. (20) identified 109 vRNA-bound proteins via a modified version of the RAP-MS protocol and linked those RBPs to RNA stability control, mRNA function, and viral process. Further, the authors showed 107 of those host factors are found to interact with vRNA of the seasonal betacoronavirus HCoV-OC43, suggesting that the vRNA interactome is highly conserved. Finally, Labeau et al. (21) used ChIRP-MS to identify 142 host proteins that bind to the SARS-CoV-2 RNA and showed, in contrast to Flynn et al. (18), that siRNA knockdown of most RBPs cellular expression leads to a significant reduction in viral particles, suggesting that the majority of RBPs represent pro-viral factors. Taken together, there is strong evidence that SARS-CoV-2, like other RNA viruses, heavily relies on the presence of a large number of essential RNA-binding host factors. However, the sets of SARS-CoV-2 relevant RBPs from different studies have limited overlap and the outcome depends on the specific cell line utilized in the experiment. While the above methods allow for the identification of RBPs that interact with SARS-CoV-2 in the context of infection, they do not give insight into where these interactions occur along the viral RNA. Indeed, a comprehensive large scale analysis of the propensities of different host RBPs to bind to RNA elements across the SARS-CoV-2 genome is currently missing. This is a severe limitation, as prevalence and high spatial resolution of protein–RNA interaction sites is necessary for understanding the hijacking of the cellular post-transcriptional machinery by the virus.

Cross-linking and immunoprecipitation (IP) followed by sequencing (CLIP-seq) assays (22), including PAR-CLIP and eCLIP protocols, are the most widely used methods to measure RBP-RNA interactions *in vivo* at high nucleotide resolution and are able to provide sets of functional elements that are directly bound by an RBP of interest (23). While CLIP-seq experiments allow for precise identification of host factor interaction with viral RNAs, the high cost of profiling interactions across a large number of RBPs becomes prohibitive at larger scales, as dedicated pull-down and sequencing has to be performed for each RBP individually. Therefore, such datasets have been generated only for a small number of proteins on SARS-CoV-2 (19). Further, in order to keep up with the continuous emergence of novel SARS-CoV-2 variants, CLIP-seq experiments would need to be repeated for the genome of each viral strain in order to account for (or to identify) gain- or loss-of-binding variants. Recent advances in machine and deep learning have enabled a cheaper but powerful alternative by computationally modeling the binding preference of RBPs using information from existing CLIP-seq datasets, such as those generated as part of the ENCODE project (24).

In this study, we present a step towards filling the gap of missing spatial information of human-SARS-CoV-2 protein–RNA interactions by predicting interaction sites computationally at nucleotide resolution. We train and optimize two recent Convolutional Neural Network (CNN) based methods, Pysster (25) and DeepRiPe (26), on hundreds of human eCLIP and PAR-CLIP datasets and use trained models to predict RBP binding on viral sequences. By that we provide, to our knowledge, the first comprehensive single-nucleotide resolution *in silico* map of host RBP-viral RNA interaction for SARS-CoV-2 as well as six other human coronaviruses and identify sequence mutations, which significantly alter RBP-RNA interaction across 11 different SARS-CoV-2 variants-of-concern. We recapitulate human RBPs, which are predicted or experimentally determined to binding to SARS-CoV-2 by previous studies and predict binding for host RBPs with no previously reported binding to SARS-CoV-2. We integrate knowledge of these proteins across other pathogens and highlight RBPs with clinical relevance, by annotating those that were found among SARS-CoV-2-associated genes from Genome Wide Association Studies (GWAS) (27), CRISPR studies (28–32), physical binding experiments (18,19,29), or patient OMICS data from blood serum and plasma (30–37). Finally, we perform extensive *in silico* single-nucleotide perturbations across the SARS-CoV-2 genome to identify mutations that would lead to gain- and/or loss-of predicted RBP binding sites and thus may alter viral fitness.

## MATERIALS AND METHODS

**Figure 1.**
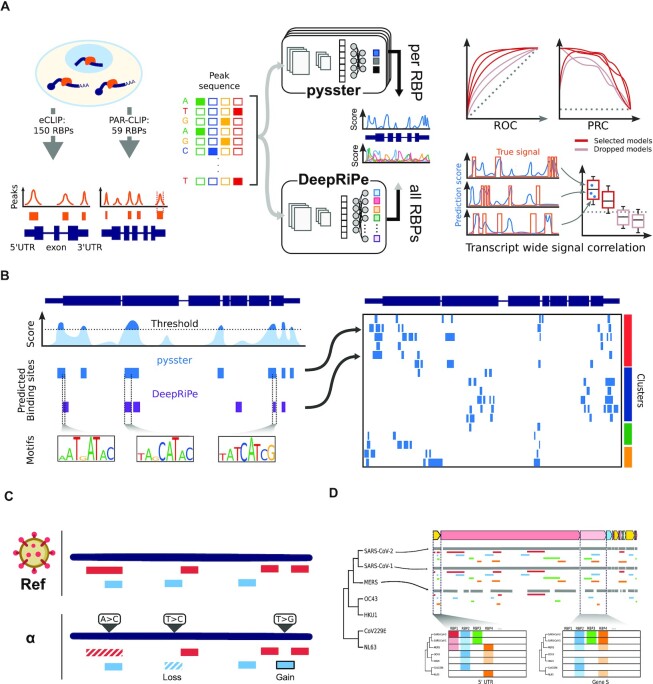
Pipeline of the computational mapping of the human - SARS-CoV-2 protein–RNA interactome. (**A**) (Left panel) Interactions between RNA-binding proteins (RBPs) and transcripts can be experimentally measured through eCLIP and PAR-CLIP protocols, enabling the quantification of locally accumulated reads, and the calling of peaks. Such peaks were obtained for 150 RBPs from eCLIP data (24), and for 59 RBPs from PAR-CLIP data (88). (Middle panel) Sequences from these peaks were used to train two deep learning models, composed of convolutional neural networks enabling the detection of complex sequence motifs. These models can then be applied to predict for a given sequence its potential for binding by a RBP. The pysster models are trained separately for each RBP, while DeepRiPe is trained in a multi-task fashion and simultaneously for all input RBPs. (Right panel) A selection of high-performance models was established through evaluation of performance of the models, from overall performance metrics to in-practice, sequence-wide evaluation. (**B**) All retained models were applied to scan the entire genome of SARS-CoV-2, and binding sites were predicted from consecutive, high-prediction scores positions. Sequence motifs underlying predicted RBP binding sites were also identified by interrogating both CNNs via Integrated Gradients. Predictions were compiled in the first *in silico* map of host-protein−viral RNA interactome for SARS-CoV-2. (**C**) The prediction models were applied to evaluate the impact of mutations from variants of concerns, (**D**) as well as to evaluate the evolutionary trajectory of affinity of host RBPs to other coronaviruses’ genomes.

The overall workflow of our approach is summarized in Figure 1, from model training, to the *in silico* mapping of the SARS-CoV-2 RBP-RNA interactome and downstream analysis. We first obtained binding site information of publicly available eCLIP experiments of 150 RBPs from the ENCODE (24) database and pre-processed them to obtain a set of high-quality sites of protein–RNA interaction. For each RBP, a convolutional neural network (CNN) classifier to predict the likelihood of RBP-binding to an arbitrary input RNA sequence was trained using the *pysster* (25) framework, resulting in 150 pysster models (Figure 1A). For RBPs not contained in the ENCODE dataset, we included DeepRiPe (26) models pre-trained on 59 PAR-CLIP datasets. Next, we performed extensive model performance evaluation on custom trained pysster models and removed poorly performing models from downstream analysis.

Using high-quality models, we predicted the probability of each RBP binding to individual nucleotides in the SARS-CoV-2 genome using a sliding-window scanning approach (Figure 1B). In addition, we controlled for false positive hits by computing, for each RBP and each single-nucleotide binding prediction, an empirical p-value using a randomized background sequence and retained only significant hits. Subsequently, consecutive high-scoring and significant positions were aggregated into larger binding-site regions. We thus constructed a comprehensive *in silico* binding map of human RBPs on the SARS-CoV-2 genome and clustered predicted RBP binding sites across different viral genomic regions to unravel potential regulatory patterns (Figure 1B). Exploiting the capability of CNNs to learn complex sequence patterns, we identified known binding motifs at predicted RBP binding sites. Finally, we utilized our models to score the impact of sequence variants identified in 11 viral variants of concern (Figure 1C) and identified conserved and novel predicted binding sites across 6 other coronaviruses, including SARS-CoV-1 and MERS (Figure 1D).

### ENCODE data and preprocessing

Enhanced CLIP (eCLIP) datasets were obtained from the ENCODE project database, which comprises 223 eCLIP experiments of 150 RBPs across two cell lines, HepG2 and K562. For RBPs with experiments in both cell lines, we selected only data of eCLIP experiments from the HepG2 cell line for downstream analysis. Narrow peaks of each eCLIP library were taken directly from ENCODE and preprocessing was performed as follows: for each of the two replicates of a given eCLIP experiment, peaks were first intersected with mRNA locations obtained from the GENCODE database (Release 35) and only overlapping peaks were retained. Next, the 5′-end of each peak was defined as the cross-linked site, as it usually corresponds to the highest accumulation of reverse transcription truncation events. A 400nt window was then centered around the cross-linked site for each peak, defining the input window of the downstream model. Input windows of both replicates were intersected reciprocally with a required overlap fraction of 0.75, ensuring that only those peaks which are present in both replicates are considered for downstream training set construction. Finally, the top most 50 000 windows with a read-start count FC of 2.0 above the control (SMInput) experiment were selected for each RBP.

### Pysster training set construction

For each RBP, a classification dataset of bound (positive) and unbound (negative) RNA sequences was constructed. Positive samples were obtained by taking corresponding 400nt peak-region windows from the previous step, while two distinct sets of negative samples were generated. First, 400nt long regions which did not overlap with CLIP peaks of the given RBP were sampled from transcripts harboring at least one CLIP peak. This constraint ensures that the transcript is expressed in the experimental cell type and would not be observed as RBP-binding in other cell types. The second set of negative samples was generated by randomly sampling CLIP peaks of other RBPs. This ensures that any CLIP-seq biases (such as U-bias during UV-C cross-linking (38,39)) are present in both positive and negative samples and prevents the model from performing a biases-based sample discrimination during the training. Together, this yields a three-class training set, where class 1 corresponds to positive samples and class 2 and 3 correspond to negative samples. Samples of class 2 and 3 were sampled at a 3:1 ratio with respect to class 1. Finally, generated samples were randomly split into train, validation and test sets at a ratio of 70:15:15, respectively.

### Pysster model

The *pysster* Python library (25) was used for implementation of the model which consists of three subsequent one-dimensional convolutional layers, each with 150 filters of size 18, followed by a single fully connected layer with 100 units. The ReLU (Rectified Linear Unit) activation function is applied to each intermediate layer output and a maximum pooling layer is added after every convolutional layer. Finally, a fully connected layer with 3 units, one for each of the three output classes, is added. Dropout (40) with a rate of 0.25 was applied to each layer, except for input and output layers. The model was trained with the Adam optimizer (41) using a batch size of 512 and a learning rate of 0.001. For each RBP, we trained for at most 500 epochs and stopped training in case the validation loss did not improve within the last 10 epochs.

### Pysster binary classification threshold

As pysster models are trained as a 3-class classification problem with class imbalance, we re-calibrated each model for the binary classification task by introducing a binary decision threshold *t*_*m*_ on the predicted positive-class probability scores. For each model *m*, *t*_*m*_ is defined as the threshold which maximized the F1 performance of the model with respect to bound vs. unbound binary classification obtained by pooling class 2 and 3 samples into a common ‘unbound’ class. This threshold is used to identify bound regions in the viral sequence.

### DeepRiPe model

To extend the set of RBPs explored, we obtained pre-trained DeepRiPe models from Ghanbari *et al.* (26) and retained models for 33 out of the 59 RBPs, filtering out models where no informative sequence motif could be learned by the model. The PAR-CLIP-based models used in this study are modified versions of the DeepRiPe neural network, where only the sequence module to extract features from the RNA sequence is used. Briefly, the model consists of two convolutional layers, one fully connected layer and one output layer that contains *k* sigmoid neurons to predict the probability of binding, one for each RBP. Each convolutional layer has a rectified linear unit (ReLU) activation, followed by a max pool layer and a dropout layer with probability of 0.25. 90 filters with length 7 and 100 filters of length 5 were used for the first and second convolution layers, respectively. The fully connected layer has 250 hidden units and a ReLU activation. Details in data preparation and model training are outlined in Ghanbari et al. (26).

### Single-nucleotide predictions

The pysster and DeepRiPe positive-class prediction score corresponds to the probability that the input RNA sequence is bound by the RBP of interest. By design, this score is assigned to the entire input sequence, although RBP binding sites are much more local, usually spanning only a few nucleotides (42). To predict single-nucleotide binding site probabilities from both pysster and DeepRiPe models along a RNA sequence, we employed a one-step sliding-window approach to scan over a given RNA sequence, where the predicted positive-class probability score is assigned to the center nucleotide of the input window. In order to obtain predictions over the entire RNA sequence, the 5′ and 3′ sequence ends were 0-padded.

### Pysster performance evaluation and model selection

As the validation loss was monitored for the purpose of early-stopping, the precision-recall (PR) and F1-score performance of the pysster models was evaluated on the test set. Models with an area under the PR curve (auPRC) of less than or equal to 0.6 were deemed poor quality and thus excluded from the downstream analysis.

Training datasets were sampled at a fixed positive-negative ratio which hardly reflects the ratio of bound and unbound sites of RNA transcripts found *in vivo*. In practice, we expect that for some transcripts regions, binding sites of a particular RBP to be not observed over several kilo-bases, while other regions, such as 5’ and 3’ untranslated regions (UTRs), might harbor a dense clustering of binding sites. To measure the ability of pysster models to accurately predict *de novo* RBP binding-sites along whole-length RNA transcripts, we introduced the concept of Performance-In-Practice (PIP), which measures how well the single-nucleotide prediction score of the model correlates with experimentally identified CLIP-seq peaks. For a given RNA sequence, the PIP of a model is defined as the Spearman correlation coefficient (SCC) between the truncated prediction scores $p_{i}^{trunc}$ and a binary vector obtained by labeling all positions that fall within eCLIP peaks with 1 and 0 otherwise. Here, $p_{i}^{trunc}$ refers to a modified version of the prediction score *p*_*i*_ defined as

$$\begin{equation*} p_{i}^{trunc}= \left\lbrace \begin{array}{@{}l@{\quad }l@{}}p_{i},& {\rm if } p_i \ge t_m\\ 0, & {\rm otherwise} \end{array}\right. \end{equation*}$$

where *t*_*m*_ is a threshold obtained for each model. For each model, we performed extensive PIP analysis on the human transcriptome as follows. First, we selected the set of transcripts which contain at least one eCLIP peak for the given RBP. From this set, we uniformly drew 100 transcripts without replacement as hold-out transcripts. Subsequently, we intersected positive and negative training samples with the hold-out transcripts and discarded all samples that overlap with any of the hold-out transcripts before retraining pysster on the remaining training samples. We used the resulting models to predict along the hold-out transcripts and compute the PIP score for each hold-out transcript. Finally, models with a median PIP score of less than or equal to 0.1 were excluded from downstream analysis.

### Comparison of pysster models with ENCODE eCLIP-based DeepRiPe models

We evaluated the correlation of prediction scores from pysster and DeepRiPe models, trained on data derived from the same ENCODE eCLIP experiments, on the SARS-CoV-2 RNA. To this end, we gathered pre-trained eCLIP DeepRiPe models from (26) and intersected them with high-confidence pysster models, yielding a total overlap of 53 RBPs. Subsequently, we performed scanning (Methods) and, for each RBP, computed the correlation (PCC) of position-wise prediction scores between the pysster and DeepRiPe models.

### Estimating significance of prediction scores

To directly control the false positive rate of predicted single-nucleotide binding scores from both pysster and DeepRiPe models on the viral genome, we estimated prediction score significance via a RNA sequence permutation test. In order to obtain a null-distribution of predictions (positive-class) scores, we first computed the di-nucleotide frequencies on the viral RNA. Next, we performesd frequency-weighted sampling of di-nucleotides to construct a set of *N* = 10 000 null-distributed inputs. Null-distributed prediction scores for each model were then obtained by predicting on those sequences. A p-value is assigned to each observed prediction score *p*_*i*_ in the viral sequence by computing the fraction of scores from the null distribution $p^{null}_j$ for which $p^{null}_j >p_i$, *j* = 1, ..., *N*.

### Predicting RBP binding sites

We predicted RBP binding sites on the viral RNA sequence using predicted single-nucleotide binding scores together with estimated p-values. For each pysster model, we classified nucleotides in the viral RNA as ‘bound’ if the predicted probability score was equal or greater than the estimated binary threshold *t*_*m*_ and the score was found to be significant (*P* < 0.01). Regions with a consecutive stretch of bound nucleotides of at least length 2 were then defined as a predicted RBP binding site. Neighboring predicted binding sites that are spaced by <10 nucleotides were merged to a single predicted binding site. Note that for DeepRiPe models, nucleotides in the viral RNA were considered ‘bound’ if the probability score is found to be significant (*P* < 0.01) and no score threshold was applied.

### Base-wise feature attribution via Integrated Gradients

To gain insight into which RNA sub-sequences are driving factors for RBP binding, we compute sequence importance scores using Integrated Gradients (IGs) (26,43). Starting from an input baseline, IG performs a step-wise linear path interpolation between the baseline and the actual input sequence and computes the gradients of the interpolated inputs with respect to an output neuron. That is, we obtained a vector of importance scores over the input sequence which indicate which nucleotides of the input contributed most toward the prediction. Here, we chose the 0-vector (i.e. the one-hot encoding of all nucleotides is set to 0) as the baseline and performed 50 baseline-input interpolation steps. To obtain sequence importance scores for a given predicted binding site, we computed IGs with respect to an input window centered around the predicted binding site. For sequence-motif construction, the heights of nucleotides in the input sequence was given by the feature attribution weights.

### Analyzing mutations in variants of concern

Mutation information of 11 SARS-CoV-2 viral variants of concern (alpha, beta, delta, epsilon, eta, gamma, iota, kappa, lambda, mu, omicron) was obtained from the UCSC genome-browser for the SARS-CoV-2 virus (44), and converted into VCF format. For each variant of concern, we first created a ‘mutated’ genome, using the viral reference sequence and the set of variant of concern specific mutations. We then centered a window at the reference position of each mutation and extracted the mutated sequence for subsequent prediction via each model. We noted that for cases where mutations were in close proximity with each other, extracted windows might contain multiple mutations. This is crucial, as only their combination might lead to gain- or loss-of-RBP-binding. The resulting prediction score on each alternative allele (ALT) was then compared with the prediction score of the same window on the reference sequence (REF). To quantify the impact of each mutation, we compute a delta score between the prediction score of ALT and REF sequence:

(1) $$\begin{equation*} \Delta _{score} = score_{ALT} - score_{REF}. \end{equation*}$$

Mutations with a positive delta score sign represent ‘gain-of-binding’ (GOB) events, while mutations with negative sign represent ‘loss-of-binding’ (LOB) events. To further narrow down the set of mutations, we compiled a subset of mutation that lead to a gain- or loss-of-binding (GOB and LOB), defined as instances where (in case of LOB) the REF score is passing the binding score threshold and p-value while the ALT does not, or vice versa (in case of gain-of-binding).

### *In silico* mutagenesis

While many mutations have been observed across sequenced samples during the pandemic, some mutational events have not yet occurred, and their impact on the fitness of the virus is thus unknown. To fill this gap, we performed *in silico* probing of the effects of all possible point-mutations on RBP binding across the SARS-CoV-2 genome. At each viral genome position, the reference base was mutated to each of the three alternative bases. Subsequently, prediction was performed on the input windows derived from each ALT allele using all high-quality pysster models. Finally, an impact score was computed and a set of change-of-binding mutations is compiled.

### Comparative analysis of human coronaviruses

Besides SARS-CoV-2, we obtained reference sequences for 6 other human coronaviruses, including SARS-CoV-1, MERS, HCoV-229E, HCoV-HKU1, HCoV-NL63 and HCoV-OC43 from NCBI (45). Using high-quality models from both pysster and DeepRiPe, we performed single-nucleotide binding prediction along each viral RNAs. Next, we computed prediction empirical p-values for each viral sequence by generating a dedicated null distribution of scores for each virus and RBP. RBP binding sites across viruses were then predicted as described previously. We evaluated genomic-element preference across a subset of shared viral genomic locations (ORF1ab, E, N, M, S, 5′ UTR, 3′ UTR) for each RBP and virus by intersecting the predicted set of binding sites of each virus with its RefSeq annotations. To compute multiple sequence alignments (MSA) between genomic elements of coronaviruses, we used the ClustalO (46) algorithm with default parameters.

### Functional annotation of RBPs

To assess the potential role of RBPs with predicted binding on viral RNA sequences, we manually curated all RNA-related functions of the 88 RBPs with good predictive models using the GeneCards, Uniprot and RBP2GO databases (47).

### Public COVID-19/coronaviruses OMICS data

To assess regulatory information of RBPs across available coronavirus/COVID-19 multiOMICS data, we downloaded evidence from 22 studies. We imported study-relevant supplementary tables via knowing01 (48), which harmonizes data tables and links results to molecular information, like human gene symbols, UniProt identifier, variant positions as available in the proprietary CellMap unified data model (Version 2022/03). After loading the 88 RBP names with high-confidence models, a list of 85 Gene Symbols with associated relevant annotations was returned. Two RBPs were dropped, namely DND1 and SRRM4, due to absence of associations. In addition, L1RE1 and ORF1 matched the same gene and thus the corresponding RBP was reported only once. To ensure that all RBP human gene symbols are identically named in African Green Monkey OMICS data, we used VeroE6 cells linked to human symbols.

A total of 97 research results were gathered and organized into six groups of evidence. The first group corresponds to RBP-SARS-CoV-2 interactomes established from affinity purification and mass spectrometry (18,19,29) and computational predictions in UTRs and Spike viral regions from catRAPIDomics (49) and PRISMNet (50). The second group gathers results from viral-host protein–protein interactions (PPIs) measured by affinity-purification followed by mass spectrometry (7,28)] and yeast two hybrid screenings (51). The third group corresponds to functional evidence from multi OMICS data, including the regulation of the host proteomics, phosphoproteomics, ubiquitinomics and transcriptomics up to 24 h after coronavirus infection (7,52), as well as the effectome, which includes deregulated host proteins 72 h after SARS-CoV-2 induced expression of each of the viral proteins (7). The fourth group includes multiomics data on SARS-CoV-1, for comparison. The fifth group includes CRISPR screens which probe cell survival few days after viral infection with single genes knockouts in human (28,53–55) or African green monkey [(9)] cell lines. Finally, the sixth group gathers data related to patients : genome-wide association studies (GWAS) linking human genetic variation to COVID-19 disease severity (27), and patient OMICS data, including proteomics and transcriptomics regulation of whole blood, serum or plasma of mostly inpatients (30–37).

To retrieve RBPs from these studies, we used the adjusted *P*-values and other data-specific cutoffs provided by each study, whenever this information was available, in order to report only significant hits. Even if the criteria used to identify significant hits were different from one study to another, most studies, e.g. interactomes of PPI networks, usually provide a core set of identified proteins, where strict p-value cutoffs were applied (e.g. FDR ≤ 0.05 for both Schmidt *et al.* (19) and Flynn *et al.* (18)), as well as expanded sets where cutoffs to identify significant hits were relaxed (e.g FDR ≤ 0.2 for Schmidt *et al.*). For data integration, we provide both hits identified at stringent cutoffs, as well as hits identified at lax cutoffs from the corresponding studies. On the OMICs data, we report a stringent cutoff (adjusted *P*-value < 0.01) and a lax one (adjusted *P*-value < 0.1), whenever available. Few data sets only provided raw *P*-values (7,30,32,34,52) for which we used a lower cutoff of *P*-value <1e−4. Patient transcriptomics data was also used at the stringent cutoff of *P*-value <1e−4, due to the inflation of regulated genes on typical cutoffs. For GWAS data we employed a genome-wide (*P*-value < 5e−08) and nominal (*P*-value < 0.01) significance cutoff, for stringent and lax cutoffs, respectively. Finally, we annotated 85 RPBs with regulated molecules and visualized the number of evidences of RBPs in each data set in a count matrix.

### Statistical analysis of RBP-binding on SARS-CoV-2

A permutation test was performed to identify human RBPs with a significant enrichment of predicted binding sites on the SARS-CoV-2 RNA. To this end, di-nucleotides of the SARS-CoV-2 RNA sequences were shuffled to generate 100 randomized viral sequences. Subsequently, binding site prediction was performed on each randomized sequence for each high-confidence human RBP model and the numbers of predicted binding sites were recorded. Using the thus generated null distribution of predicted binding site counts, a *P*-value was computed for each RBP as the number of times a randomized sequence harbored more predicted binding sites than the true SARS-CoV-2 RNA sequence, divided by the number of randomized sequences (100). A pseudo-count was added to prevent *P*-values of 0. Finally, we corrected for multiple testing by adjusting *P*-values following the Benjamini-Hochberg procedure and significantly enriched RBPs were selected using a FDR threshold of 0.1.

### Statistical analysis of SECReTE-Motifs

To estimate whether clusters of RBPs show a significant enrichment of binding sites overlapping with SECReTE motifs on the SARS-CoV-2 RNA, we performed a Fisher exact test of each cluster. Specifically, a contingency table is constructed with (i) number of cluster binding sites overlapping with SECReTE motifs, (ii) number of non-cluster binding sites overlapping with SECReTE motifs, (iii) number of cluster binding sites not overlapping with SECReTE motifs and (iv) number of non-cluster binding sites not overlapping with SECReTE motifs. To obtain p-values, we took the sum over the upper tail of the hyper-geometric distribution. Finally, we accounted for multiple testing by adjuting *P*-values with the Benjamini-Hochberg correction.

## RESULTS

### Accurate model predictions in human and viral sequences

**Figure 2.**
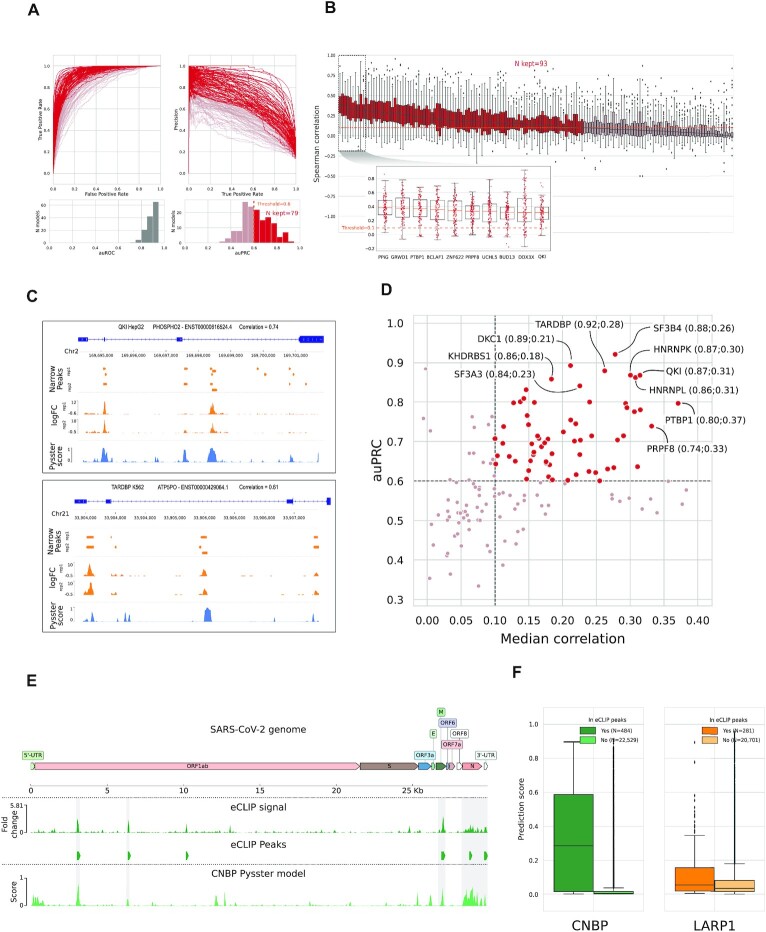
Evaluation of pysster models’ performance and high-quality model selection. (**A**) Receiver Operating Curve (ROC) and Precision Recall Curve (PRC) for all 150 pysster models trained from ENCODE eCLIP datasets. A first threshold of 0.6 was set on the area under the PRCs (auPRC), leading to a subset of 79 models passing the threshold. (**B**) Boxplots of correlations between eCLIP and prediction scores from 100 left-out transcripts per RBP model. This correlation highlights the performance of models in a realistic context of full-sequence-length scan. A second threshold was thus set on the median correlation coefficient, leading to a subset of 93 models passing the threshold. The 10 models with highest median correlation are displayed in a detailed sub-plot. (**C**) Genome-browser view illustrating the comparison between eCLIP signals and model prediction scores over full-length transcripts. Two of the best models are presented, with signal from left-out transcripts with high correlation between eCLIP log-fold-change signals and prediction scores from the pysster models. (**D**) Scatterplot of the AUPRC and median correlation values for each model, highlighting the final subset of high-quality models. The top 10 models are labeled. (**E**) Comparison of genome-wide eCLIP signal and pysster prediction scores from the CNBP eCLIP datasets generated over the SARS-CoV-2 genome by (19). (**F**) Boxplot of pysster prediction scores from position within or without overlap from called narrow peaks, for the CNBP model and the LARP1 model (*t*-test *P*-values: < 1e−16 for CNBP; 2.44e−6 for LARP1).

The trained pysster models showed a robust area-under-precision-recall-curve (auPRC) performance, with a median auPRC of 0.6 across all 150 trained models (Figure 2A, Supplementary Table S1). As models were used for scanning of the full-length viral genome (rather than classification of standalone examples), we further evaluate the model performance by computing the correlation of the predicted positive-class probabilities with observed ENCODE peaks on a hold-out set of human transcripts. Nearly all models showed a significant positive correlation, with a mean median Spearman correlation coefficient (SCC) across transcripts of 0.149 and a maximum median SCC of 0.38 (Figure 2B, Supplementary Table S1). Considering that eCLIP experiments have a relatively low signal correlation baseline between replicates (56), this indicates that the trained models are well-suited for the task of scanning across the viral genome. Exemplary prediction tracks for two held-out human transcripts using pysster models of QKI and TARDBP are shown in Figure 2C. In general, we observe that models which perform well with respect to the auPRC score tend to perform well in the context of RNA sequence scanning (Figure 2D). To ensure that downstream analyses are based on a high-quality set of binding site predictions, models with a median SCC of < 0.1 or an auPRC of < 0.6 were discarded. A total of 63 high-quality pysster models were thus kept for predicting on the SARS-CoV-2 genome. For DeepRiPe, we relied on the results from (26) and retained only those models where informative sequence motifs were learned during training, leaving a total of 33 RBP models for predicting on the SARS-CoV-2 genome. Of those, we selected only models for RBPs not contained in the ENCODE database, leading to the addition of 24 high-quality DeepRiPe models.

The lack of eCLIP data in a SARS-CoV-2 infection context prevents a large-scale performance evaluation of trained pysster and DeepRiPe models directly on the SARS-CoV-2 RNA sequence. Nevertheless, we obtained eCLIP data for two proteins, CNBP and LARP1, from Schmidt *et al.* (19), allowing us to partially evaluate the validity of our cross-species (human/virus) training and prediction approach. After generating training samples from CNBP and LARP1 eCLIP peaks within human transcripts (processed in the same manner as eCLIP peaks from the ENCODE experiments), we trained pysster models for both RBPs (Supplementary figure 1A). We then performed prediction along the SARS-CoV-2 RNA sequence and compared the resulting single-nucleotide prediction scores with observed eCLIP peaks as well as the eCLIP signal provided by the authors (Figure 2E and 2F, Supplementary Figure 1C). Predictions showed an auPRC of 0.72 and 0.56, respectively, and a strong correlation with the raw eCLIP signal (SCC = 0.332, *P*-value < 1e−16 for CNBP and SCC = 0.133, *P*-value = 7.96e−12 for LARP1). In addition, we observed an accumulation of high-scoring positions at the location of experimentally identified peaks for CNBP and (to lesser extent) LARP1 (Figure 2F, Supplementary Figure 1C). We therefore excluded the LARP1 model from the final set of models, as it does not meet the established performance criteria (Methods), while the CNBP model was retained. Although it is difficult to assess to what extend these results generalize to other RBPs, high performing models selected in the previous section are likely suitable for cross-species, *in silico* prediction of RBP binding sites on SARS-CoV-2.

### A comprehensive *in silico* binding map of human RBPs on SARS-CoV-2

We performed *in silico* binding site prediction by identifying consecutive significant and high-scoring positions within the SARS-CoV-2 genome with both pysster and DeepRiPe high-confidence models (Methods). In the following, we first demonstrate that our model makes predictions on the basis of genuine sequence features and subsequently build a computational map of predicted SARS-CoV-2-human RBP interactions. We then evaluate the enrichment of different RBPs for different viral genomic regions, as well as their putative regulatory function in the context of SARS-CoV-2 infection.

**Figure 3.**
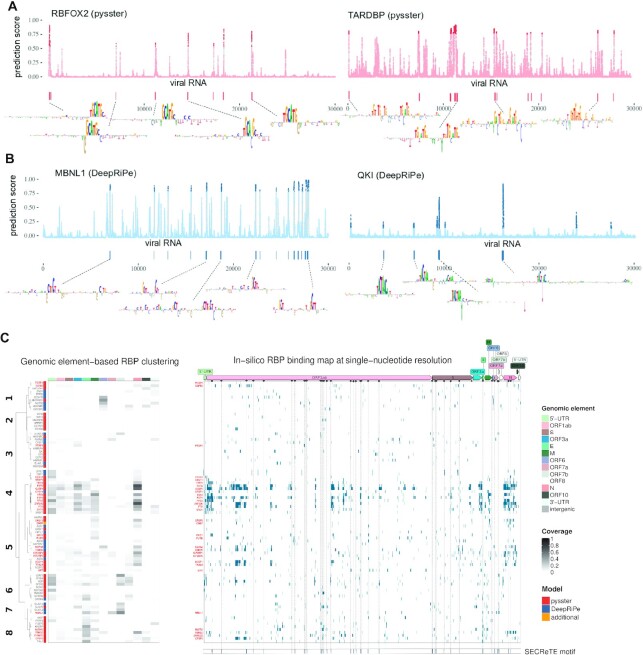
Computational map of RBP binding on SARS-CoV-2. (**A**) Single-nucleotide probability score for RBFOX2 (left) and TARDBP (right) RBP binding as computed by the corresponding pysster models across the whole SARS-CoV-2 genome. The higher the score, the higher the likelihood of a binding event at that position. Points highlighted in strong color correspond to significant predictions, i.e. with bound probability significantly higher than random (empirical p-value < 0.01, see Methods). Wider predicted binding sites, encompassing more than one significant position are shown as vertical bars underneath each prediction profile, together with their corresponding binding motifs as extracted by means of attribution maps (see Methods). (**B**) Single-nucleotide probability score for MBNL1 (left) and QKI (right) RBP binding as predicted by the corresponding DeepRiPe models. Significant positions (empirical *p*-value < 0.01) are highlighted in strong color, and predicted binding sites together with their corresponding motifs are shown underneath. (**C**) Clustering of RBPs based on predicted binding site coverage of genomic annotations of SARS-CoV-2 for both pysster and DeepRiPe RBPs (left panel). *In silico* RBP binding map, at single-nucleotide resolution, for both pysster and DeepRiPe RBPs (right panel). RBP names in bold and red indicate those with statistically significant number of predicted binding sites when compared against predictions in 100 shuffled genomes, see methods. SARS-CoV-2 SECReTE motifs from (67) are shown below, with vertical lines helping the visualization of overlaps with predicted binding sites.

**Figure 4.**
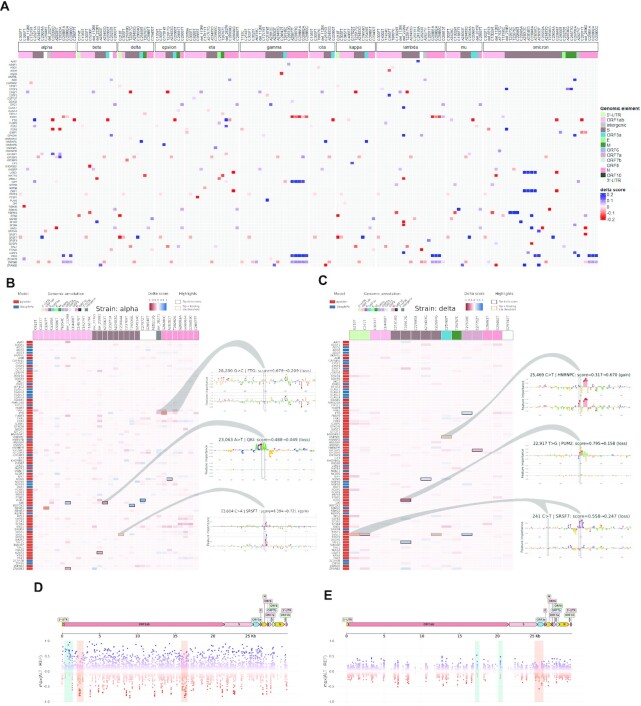
Impact of mutations from SARS-CoV-2 variants of concern on predicted binding sites. (**A**) Joint heatmap of delta scores from the 290 identified mutations in the different SARS-CoV-2 variants of concern. Delta scores represent the difference in prediction score of a prediction model between alternative and reference sequences centered on each mutation. Only the 315 impacts labeled as change-of-binding are colored. Delta score color scale is capped so as to show low delta score impacts. RBPs and mutations without any such impact across strains are dropped from the heatmap. (**B**) Complete heatmap of delta scores from 31 mutations associated to the alpha viral variant. The top 10 with highest absolute delta scores are lined out, with yellow color indicating the ones labeled as change-of-binding. Some sites are further investigated through integrated gradients, comparing the sequence motifs identified by the prediction models against known motifs from mCrossBase (89). (**C**) Complete heatmap of delta scores from 16 mutations associated to the delta viral variant. (**D**, **E**) Results from the *in silico* mutagenesis over the SARS-CoV-2 genome for PUM2 and FTO, respectively. Nucleotides across the viral genome were perturbed towards the three alternative bases, generating a reference distribution of possible delta scores, notably highlighting positions with highest impacts. Highlighted regions show hypothetical variants that are predicted to lead strong gain- (blue) or loss- (red) of-RBP-binding.

**Figure 5.**
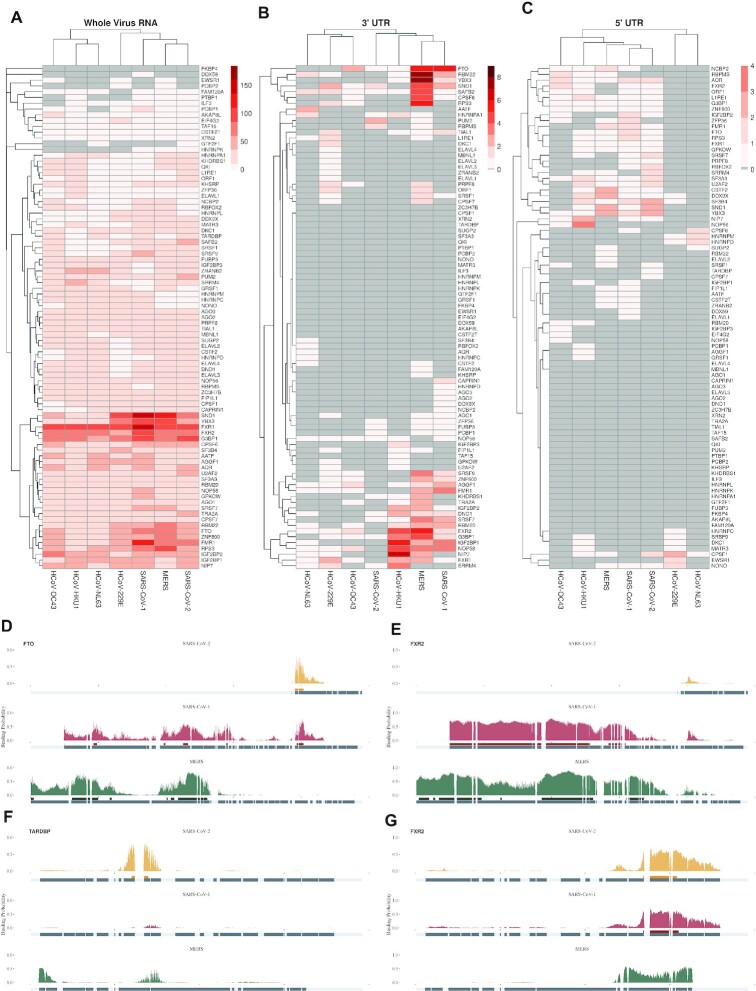
Comparison of SARS-CoV-2 and 6 other human coronaviruses. (A−C) Binding sites were predicted over the seven human coronaviruses, and their number counted over the entire genome (**A**) or over the 3’ (**B**) and 5’ (**C**) UTRs. Hierarchical clustering was applied to evaluate the proximity between viruses in terms of predicted binding sites composition. (D−G) Examples of evolutionary conserved, gained, and lost binding sites between the three high-severity viruses MERS, SARS-CoV-1, and SARS-CoV2. Panel **D** shows an example for predicted FTO binding sites found only in SARS-Cov-2 and SARS-CoV-1 in their 3’ UTRs. Panel **E** shows a predicted binding site for FXR2 only shared between MERS and SARS-CoV-1 in their 3’ UTR. Panel **F** shows a predicted binding site for TARDBP exclusive to SARS-CoV-2 in the 5’ UTR. Panel **G** shows a predicted binding site for FXR2 only shared between SARS-CoV-2 and SARS-CoV-1 in the 5’ UTR.

**Figure 6.**
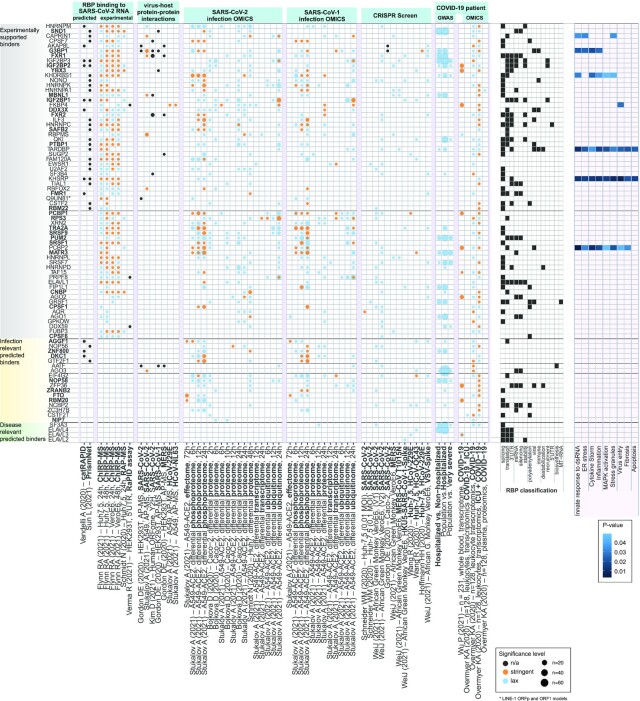
RBPs in context of public *in vitro* and patient OMICS data. RBP with model predictions (rows) annotated with experimental evidences found in 92 multiOMIC publicly available research results (columns) followed by information from RBP classification and role in known SARS-CoV-2 pathways. From left to right: RBPs were manually assigned to three categories according to their annotation pattern. RBPs predicted to bind SARS-CoV-2 RNA by the other prediction methods catRAPID, PrismNET. RBPs binding to SARS-CoV-2 RNA determined experimentally by ChIRP-MS, RAP-MS and RaPID assay. Evidences of RBPs with stringent or lax significance cutoffs found in further 55 data sets across multiple OMICS levels and experiment types were grouped by experimental context: Experimental viral-host protein interactions measured by AP-MS across various coronaviruses, SARS-CoV-2 and SARS-CoV-2 infection OMIC (timecourses), selected CRISPR studies, most recent GWAS data (release 6) by Host Genetics Initiative and blood-based patient OMICS data. Classification of RBP according to their roles related to biological processes. Far right: Annotation of RBPs to pathways related to SARS-CoV-2 infections obtained from SIGNOR database.

**Table 1.** Subset of high delta score mutations passing binding sites thresholds

	RBP	Variant	Strain	Genomic element	REF score	ALT score	delta score	Impact
0	SRSF7	G210T	delta, kappa	5’ UTR	0.768	0.457	−0.311	loss
1	RBM20	C3267T	alpha	ORF1ab	0.813	0.336	−0.477	loss
2	RBM22	C18877T	mu	ORF1ab	0.338	0.614	0.276	gain
3	HNRNPC	C21575T	iota	S	0.374	0.840	0.467	gain
4	MBNL1	del_22281	beta	S	0.800	0.006	−0.795	loss
5	ELAVL1	del_22299	lambda	S	0.070	0.632	0.562	gain
6	SF3B4	del_22299	lambda	S	0.871	0.128	−0.744	loss
7	SF3A3	del_22299	lambda	S	0.860	0.273	−0.587	loss
8	U2AF2	del_22299	lambda	S	0.543	0.980	0.438	gain
9	GPKOW	del_22299	lambda	S	0.297	0.841	0.544	gain
10	MBNL1	del_22299	lambda	S	0.803	0.398	−0.405	loss
11	SF3A3	C22995A	omicron	S	0.081	0.808	0.726	gain
12	ORF1	A23013C	omicron	S	0.014	0.621	0.608	gain
13	ORF1	A23040G	omicron	S	0.006	0.673	0.666	gain
14	ORF1	G23048A	omicron	S	0.006	0.606	0.600	gain
15	SND1	G23048A	omicron	S	0.187	0.791	0.604	gain
16	SRSF7	C23604A	alpha, mu	S	0.394	0.719	0.326	gain
17	SRSF7	C23604G	delta, kappa	S	0.394	0.792	0.398	gain
18	HNRNPC	C25469T	delta, kappa	ORF3a	0.317	0.670	0.352	gain
19	FTO	G25563T	beta, epsilon, iota, mu	ORF3a	0.633	0.080	−0.552	loss
20	FTO	del_28278	eta	N	0.335	0.683	0.348	gain
21	FTO	G28280C	alpha	N	0.679	0.209	−0.470	loss
22	ORF1	A28699G	eta	N	0.597	0.141	−0.456	loss

#### Predicted RBP binding sites coincide with known binding motifs

While deep neural networks yield high performance on the task of protein–RNA interaction prediction, they are often considered to be black-box predictors, with predictions generally difficult to interpret. Nevertheless, it is imperative to assert that the model’s binding predictions are performed due to presence of genuine RNA sequence features associated with protein–RNA interaction, rather than spurious signal incorporated into the model due to technical biases in the training set. As only a fraction of RBPs are known to bind to distinct primary motifs, we performed feature importance analysis for selected RBPs in order to assess whether the sequence features underlying predictions at predicted binding sites correspond to the binding site preferences of those proteins reported in literature.

Figure 3A and 3B each show single-nucleotide resolution prediction scores of the human RBPs RBFOX2 and TARDBP, obtained from pysster models, and MBNL1 and QKI, obtained from DeepRiPe models. Predicted binding sites are shown below the prediction score tracks. We centered input windows around predicted binding sites of RBFOX2, TARDBP, MBNL1 and QKI on SARS-CoV-2 to identify individual nucleotides that were most predictive for classifying the input sequence as ‘bound’ (Figure 3A and 3B; bottom track). We observed that feature importance maps around predicted binding sites corresponded to known binding motifs. For instance, we observe the well-known consensus sequence (T)GCATG recognized by the splicing factor RBFOX2 (57) in the corresponding feature importance maps (Figure 3A, left), as well as the TG-repeat motif, corresponding to the sequence preference of TARDBP (58), coinciding with its predicted binding sites (Figure 3A, right). Similarly, DeepRiPe attribution maps with respect to binding sites of QKI show the canonical binding motif TACTAA(C) (59) (Figure 3B, left). Lastly, the attribution maps computed at each predicted binding site of the splicing factor MBNL1 all harbor occurrences of the characteristic YGCY motif (60) (Figure 3B, right).

#### Binding site predictions are robust across different datasets and prediction tools

To evaluate the robustness of viral binding site predictions across pysster and DeepRiPe, we compared predictions for a small set of RBPs where both eCLIP data (used to train pysster models) and PAR-CLIP data (used for the training of DeepRiPe models) were available. (For a comparison with DeepRiPe models trained on eCLIP data, see Supplementary Table S2.) Among a total of 20 overlapping RBPs, 12 were contained in the sets of high-quality models for pysster and DeepRiPe, namely TARDBP, CSTF2, IGF2BP1, PUM2, CSTF2T, QKI, IGF2BP2, IGF2BP3, CPSF6, FXR1, FXR2 and EWSR1. For each of the 12 RBPs, we then computed the correlation between the pysster and DeepRiPe prediction scores across single-nucleotide positions on the viral genome. We observed a signal correlation higher than 0.1 for 8 out of the 12 RBPs, with a correlation coefficient ranging from a maximum of 0.64 (TARDBP) to a minimum of 0.15 (CPSF6) (Supplementary Table S3). In general, we observed a higher overlap between pysster and DeepRiPE binding site predictions for RBPs harbouring well-defined RNA sequence motifs, such as QKI, TARDBP, PUM2, CSTF2, and to a less extent, FXR1-2 and IGF2BP1-3. In addition, feature attributions maps at overlapping predicted binding sites of pysster and DeepRiPe with respect to QKI and TARDBP (Supplementary Figure S2), highlight the presence of the known binding motifs for these two RBPs.

#### Binding preferences and clusters of human RBP predicted sites on the SARS-CoV-2 genome

Given the set of 88 high confidence models comprised of pysster models trained on ENCODE eCLIP data and supplemented by DeepRiPe models trained on PAR-CLIP data, we build a comprehensive *in silico* SARS-CoV-2 / human RBP binding map. Note that eCLIP DeepRiPe models were not included here, as these models were trained with an architecture optimized for PAR-CLIP datasets (26), while in addition, high predictions correlation was observed between pysster and DeepRiPe eCLIP models for many RBPs (Supplementary Table S2).

Figure 3C (right) depicts the predicted binding profiles of 84 (out of 88) human RBPs which harbor at least one predicted binding site on the SARS-CoV-2 sequence. Among those, 31 were found to exhibit a significant enrichment of predicted binding sites on SARS-CoV-2 compared to a background of randomly shuffled sequence of the same length (see Materials and Methods, Figure 3C, Supplementary Table S4). Next RBPs were clustered into eight classes based on their relative predicted binding site coverage across different genomic regions of the SARS-CoV-2 genome (Figure 3C, left). While some clusters of proteins exhibit sparse predicted binding signal across the SARS-CoV-2 genome (such as clusters 2 and 3), other clusters contain almost exclusively RBPs with enriched predicted binding across the whole SARS-CoV-2 genome (cluster 4).

We observe overall extensive RBP binding coverage mostly at 5’ UTRs and genomic regions coding for E, M and N structural proteins, and less coverage at the spike S gene, as well as the viral 3’ UTR. Clustering of predicted binding sites groups together RBPs with similar roles in RNA processing and viral regulation, RNA recognition mechanisms and preferences for certain genomic locations. Cluster 4 showed the highest accumulation of RBPs with predicted binding enriched on the SARS-CoV-2 sequence. This clusters corresponds to a group of well-known regulators of RNA processing, including proteins from the FXR family (FXR1, FXR2 and FMR1) (23), which extensively bind the viral 5’ UTR, as well as the ORF1ab and subgenomic RNAs. Other proteins in this cluster include DDX3X, regulator of RNA translation and host target against SARS-CoV-2 infection (61), splicing regulators (SR) SRSF1 and SRSF2 and RNA demethylase FTO, implicated in HIV infection (62,63). Further, cluster 1 predominantly harbors RBPs with predicted binding preference for the viral 3’ UTR, including regulators of RNA stability and proteins involved in 3’ end formation and/or regulation of translation. Cluster 6 is comprised of RBPs which preferentially bind to the 5’ UTR of SARS-CoV-2 and are involved in splicing (23). It contains NONO, a member of the paraspeckle complex, previously found in the RBP interactome of SINV infected cells (13), as well as TARDBP, a protein that localizes to P-bodies and stress granules and was shown to bind to the 5’ UTR of SARS-CoV-2 in a recent study (64). Cluster 5 includes RBPs with diverse functions who preferentially bind to the N and M viral genomic regions, while RBPs in cluster 7 and 8 were mostly predicted to bind ORF7b, as well as E and M regions. Besides the splicing regulators MBNL1 and sUGP2, cluster 7 contains members of the ELAVL family, previously shown to be prone to be hijacked during viral infections (65). While most RBPs in cluster 8 were not found to be functionally related in literature, RBPs KHSRP and MATR3 have been shown to act as restriction factors in SINV infection (66).

#### Predicted RBP binding sites overlap with SECReTE motifs

Haimovich et al. (67) recently identified the presence of a unique *cis*-acting RNA element, termed ‘SECReTE’ motif, which consists of 10 or more consecutive triplet repeats, with a C or a U present at every third base, on the sequences of both (−) and (+)ssRNA viruses. In context of SARS-CoV-2, a total of 40 SECReTE motifs have been identified in the viral genome, with a total length of ∼1.3 kb. This motif has been suggested to be important for efficient translation and secretion of membrane or ER-associated secreted viral proteins, as well as for viral replication centers (VRCs) formation. To investigate whether predicted binding sites coincide with SECReTE motifs, we obtained exact locations of all SARS-CoV-2 SECReTE motifs from (67), and subsequently intersected them with predicted RBP binding sites of all 84 high-quality models containing at least one predicted binding site in SARS-CoV-2 (Supplementary Table S5). We observed that a total of 61 RBPs (out of 84) have predicted binding sites overlapping with SECReTE motifs. Further, 30 RBPs with at least 10% of their predicted binding sites overlapping with SECReTE motifs were identified. We next investigated whether clusters of RBPs binding to SARS-CoV-2 (Figure 3C) are enriched in binding sites overlapping with SECReTE-motifs. We find that clusters 7 and 8 show a significant enrichment (adj.*P* < 1.56e−05 and adj.*P* < 0.0667) of binding sites overlapping with SECReTE-motifs on SARS-CoV-2 genome, while clusters 1 and 5 show a significant depletion (adj.*P* < 0.0291 and adj.*P* < 0.0404) at a significance level of 0.1. For instance, cluster 8 harbors 4 (out of 9) SECReTE-associated RBPs (FUBP3, KHSRP, MATR3 and CPSF6), 3 of which (FUBP3, KHSRP, MATR3) have 25% or more of their predicted binding sites overlapping with SECReTE motifs. KHSRP is an essential RBP involved in RNA localization, RNA stability and translation, while METR3 is a regulator of RNA stability. Interestingly, most of these factors have been previously associated to viral RNA regulation (23). Similarly, all 4 RBPs in cluster 7 (ELAVL2, ELAVL3, SUGP2 and MBNL1) have > 25% of their respective predicted binding sites overlapping genomic regions harboring SECReTE motifs.

### SARS-CoV-2 variants of concern show gain- and loss-of-binding events

Multiple waves of SARS-CoV-2 infections have spread across the globe, some of which resulted in the emergence of specific lineages of viral variants. The systematic sequencing of thousands of samples from infected patients enabled the description and categorization of the detected viral sequences, identifying numerous mutations in their sequence when compared to the initial SARS-CoV-2 reference genome. Some of the thus described strains have been experimentally characterized as more efficient than others, explaining in part their successful spread at local or global geographic scales (68–70). These strains have been defined by the World Health Organization as variants of concern, with ‘evidence for increased transmissibility, virulence, and/or decreased diagnostic, therapeutic, or vaccine efficacy’ (71). Specific subsets of mutations have been associated with each variant of concern, when mutations were represented in a majority of sequenced samples of their lineage. Notably, a special focus has been given with regards to the impact of mutations occurring within the spike-encoding gene (72), owing its importance in the initial steps of viral infection and its potential for vaccine neutralization (73). However, due to a lack of appropriate methods, the impact of these mutations at the regulatory level, such as their impact on protein–RNA interactions, has so far been largely ignored. To fill this gap, we systematically investigated the impact of observed mutations in viral variants of concern on the predicted binding of RBPs, in order to uncover potential viral hijacking of host proteins directly at the RNA level.

#### A catalog of high-impacting mutations across 11 variants of concern

We compiled a total of 290 mutations (193 unique mutations, 37 shared across strains) across 11 variants of concern, including alpha, delta, and omicron strains. For each variant and RBP, we evaluated the impact of the variant in terms of gain- or loss-of-binding by comparing the predicted binding probability of the reference and alternative allele. Using pysster and DeepRiPe models across 87 RBPs, we obtained a total of 25,230 impact scores, one for each pair of variant and RBP. Notably, three variants (3,037C>T, 14,408C>T and 23,403A>G) are consistently found across all viral strains, and their highest absolute delta scores were respectively associated to FTO (avg. decrease from 0.474 to 0.356), AQR (avg. decrease from 0.191 to 0.036), and NONO (avg. increase from 0.086 to 0.340). In order to prioritize pairs of variants and RBPs that show a gain- or loss-of-binding, we select a subset of pairs for which either the reference *or* alternative allele pass our binding thresholds. Note that this filter applies a XOR operation, i.e. we are interested in events that lead to either gain- or loss-of-binding (GOB, LOB). Overall, a total of 315 GOB or LOB events passed the above filter and are depicted in Figure 4A. The majority of variants introduced small delta in prediction scores, with less than 20% (61) of absolute delta-scores above 0.233 (Figure 4A). As shown in the Supplementary Figure S3A, the top 20% highest-impact variants from Figure 4A accumulate in different genomic annotations over the SARS-CoV-2 genome. Interestingly, among the RBPs impacted by these mutations, we find that some variants of concern present multiple high delta score mutations for SRSF7 (delta, kappa) and YBX3 (lambda), as well as L1RE1, RBPMS, SND1, ZRANB2 (omicron) (Supplementary Figure S3B and S3C). Additionally, the omicron variant of concern harbors a particularly large number of variants predicted to impact binding of ORF1 protein (from LINE-1 retrotransposable element).

#### Systematic point-wise *in silico* mutagenesis reveals hypothetical high-impact variants

New viral strains are continuously emerging, some of which are characterized by a faster spread due to newly acquired sequence variants, highlighting the importance of a continuous monitoring of viral variants which may result in a selective advantage on the protein or RNA regulatory level. To anticipate and quantify the impact of potentially unobserved variants, we perform a systematic *in silico* mutagenesis by generating all possible point mutations across the SARS-CoV-2 genome and score each hypothetical mutation with respect to its impact on RBP binding. Figure 4D and 4E show exemplary *in silico* mutation tracks for PUM2 and FTO, respectively, with observed reference prediction scores depicted at the top and the impact of gain- and loss-of-binding variants shown at the bottom. Note that for visualization purpose, only the delta score of the alternative allele with the highest impact is shown for each position and RBP. Supplementary Figure S4 shows an impact catalogue of 29,903 × 63 single-nucleotide variants across all SARS-CoV-2 genome positions and 63 pysster models. Complete set of hypothetical variants together with their impact scores is available at https://sc2rbpmap.helmholtz-muenchen.de/.

#### High-impact sequence variants disrupt known RBP-binding motifs

As *in vivo* RBP-binding is usually driven via the recognition of short sequence motifs, we investigated whether high-impact variants cause gain- or loss- of known binding motifs. To this end, we gathered from each strain the top 10 variants with highest absolute delta scores, as illustrated in Figure 4B and 4C for strains alpha and delta, respectively. This represented a total of 69 unique mutation-RBP pairs, 19 of which were found in more than one strain. As expected, the majority (54/69) of their delta scores is found to be in the top 1% of the distributions from the *in silico* mutagenesis. We then computed feature attribution scores, centered at the position of each high-impact variant. Feature attribution maps for the subset of candidate high-impact variants of the alpha and delta strain are shown in Figure 4B and 4C, respectively. Indeed, we observe that variants with high negative delta score tend to disrupt known binding motifs of human RBPs. For instance, transition T>G at position 22,917, as seen in the delta strain (Figure 4C) (as well as in top mutations from epsilon and kappa strains) decreases the prediction score for PUM2 from 0.795 to 0.158, with only 0.0015% in silico variants showing a lower delta score. As is clearly visible from the feature attribution analysis (Figure 4C; middle-right), the variant disrupts the well-known PUM2 binding motif TGTATAT. In a similar manner, transversion A>T at position 23,063 from the alpha strain (Figure 4B; also found in top mutations from beta, gamma, and mu strains) decreases the prediction score for QKI from 0.488 to 0.049, with 0.006% *in silico* mutations show a low delta score. Here, the feature attribution profiles clearly highlight how the known QKI binding motif ACTAA was detected by the model in the reference sequence, and how the mutation leads to a loss of this motif. Lastly, the transversion G>C at position 28,280 in the alpha strain (Figure 4B) decreases the prediction score for FTO binding from 0.679 to 0.209, and only 6 (0.00007%) *in silico* mutations show a delta score lower than the one observed (Figure 4D). Although no clear motif is found within the window, the heights of the nucleotides at the position of the mutation are reduced compared to the reference sequence, reflecting the decreased prediction score.

#### High-impact gain- and loss-of-binding events across viral strains

Among the above set of top 10 highest impact variants per viral strain, we select those that conform to strict gain- or loss-of-binding. We identify a total of 23 (out of 69) change of binding events across 17 variants and 13 RBPs (Table 1). The first example corresponds to a transversion G>T at position 210 in the 5’UTR from the delta and kappa strains, predicted to induce a loss-of-binding for SRSF7, which we had confirmed from the loss of binding motif (delta strain heatmap, see Figure 4C). Further, from the ORF1ab gene, two examples of a loss of binding for RBM20 by the C>T transition at position 3,267 (strain alpha), and a gain-of-binding of RBM22 from a C>T transition at position 18,877 (strain mu). From the S gene, a gain-of-binding is reported for HNRNPC, induced by a C>T transition at position 21,575 (strain iota), in addition to another gain-of-binding reported for SF3A3, from a C>A transversion at position 22,995 (strain omicron). Two mutations occurring in the ORF3a gene are passing our filters for two RBP impacts: the transition C>T at position 25,469 induces a gain of binding for HNRNPC in delta and kappa strains, while the G>T transversion at position 25,563 induces a loss of binding for FTO in strains beta, epsilon, iota and mu. Finally, in the N gene, we report three mutations, two of them impacting FTO binding (one gain in the eta strain, from a deletion at position 28,278; one loss in the alpha strain, from a G>C transversion at position 28,280), and a loss-of-binding of ORF1 protein (from LINE-1 retrotransposable element) in the eta strain, from a A>G transversion at position 28,699.

#### Individual variants impact binding of several RBPs

Among variants that surpass binding sites thresholds and lead to either gain- or loss-of-binding, several variants impact RBP binding of multiple RBPs simultaneously. For instance, a deletion at position 22,299 (S gene) identified in the lambda strain, is predicted to induce a gain-of-binding for ELAVL1, U2AF2 and GPKOW, while inducing a loss-of-binding for SF3B4, SF3A3 and MBNL1. Interestingly, all these factors are associated with splicing. Notably, the MBNL1 loss is also detected in the beta strain, through a deletion happening in a close-by location (at position 22,281, S gene), suggesting those two mutations may have been retained due to beneficial induction of similar changes in binding patterns. Another mutation which impacts multiple RBPs is the transition G>A at position 23,048 (S gene) from the omicron strain, predicted to induce binding of the ORF1 protein from LINE-1 retrotransposable element, as well as of SND1. Comparably to the MBNL1 impact, two close-by mutations from omicron were associated with a gain of ORF1 binding (transversion A>C at position 23,013, and transition A>G at position 23,040), further suggesting joint impact of these mutations on ORF1p binding. The last case of mutations with impact on multiple RBPs concerns a set of 2 mutations: C>A transversion and C>G transversion at position 23,604, in the S gene. The first is found in alpha and mu strains, while the second is found in the delta and kappa strains. Both mutations are predicted to induce a gain of SRSF7 binding, which is visualized for the alpha strain on Figure 4B through feature attribution maps.

### Predicted RBP-binding across human coronaviruses

While evaluation of impact for reported variants enables the monitoring of potentially functional changes in the SARS-CoV-2 genome, evaluating changes in binding sites at longer evolutionary time scale might highlight more fundamental properties of the SARS-CoV-2 virus, as compared to other RNA viruses infecting human. We investigated to which extent predicted binding sites of human RBPs are conserved across related human coronaviruses. For this purpose, we obtained genomes and genomic annotations of 6 SARS-CoV-2-related human coronaviruses, namely SARS-CoV-1, MERS, HCoV-OC43, HCoV-NL63, HCoV-HKU1, HCoV-229E. Binding sites were predicted in analogy to SARS-CoV-2 across each viral genome using 87 high-confidence pysster and DeepRiPe models. Figure 5A shows the general predicted binding propensity of RBPs across viral genomes of the 7 coronaviruses. Overall, predicted RBP binding is conserved across coronaviruses, with the highly pathogenic viruses (SARS-CoV-1, SARS-CoV-2 and MERS) showing a highly similar predicted pattern. Further, a total of 86 (out of 87) RBPs (except FKBP4) were predicted to harbor a predicted binding site in at least one coronavirus, with only a small variability in the total number of predicted binding RBPs between individual viruses. However, we observe a greater variability of predicted RBP binding within shared genomic regions across coronaviruses, for instance in the 5’ and 3’ untranslated regions (UTRs). Viral UTRs are known to play an important role in both pro- and anti-viral responses and recent evidence suggests that evolution of the 3′ UTR is contributing to increased viral diversity (74). Indeed, the 3′ UTR of SARS-CoV-2 shows a severe truncation when compared to SARS-CoV-1 and MERS. Given that viral UTRs are not under selective pressure with respect to a translated protein, they might be more prone to acquire mutations that modulate regulation through host RBPs. Figure 5B and 5C shows predicted RBP binding sites to the 3′ and 5′ UTRs across selected coronaviruses, respectively. While SARS-CoV-1, SARS-CoV-2 and MERS show conserved predicted binding on the 5′ UTR and cluster closely, a depletion of predicted RBP binding sites is observed in the 3′ UTR of SARS-CoV-2 when compared to SARS-CoV-1 and MERS. To investigate gain- and loss-of-binding in viral UTRs across the severe pathogenic human coronaviruses SARS-CoV-1, SARS-CoV-2 and MERS, we performed multiple sequence alignment of the viral 3’ and 5’ UTRs and compared the predicted binding score profiles across the three viruses.

#### Loss of FXR2-binding in SARS-CoV-2 3′ UTR

Figure 5E shows predicted 3′ UTR binding of FXR2, a paralog of FMRP (fragile X mental retardation protein). Our model predicted extensive binding of FXR2 along the 3′ UTR of SARS-CoV-1 and MERS, while SARS-CoV-2 showed a complete lack of predicted FXR2 binding sites, owing to its significantly shorter 3′ UTR. On the other hand, Figure 5G shows that predicted FXR2 binding is conserved in the 5’ UTR of SARS-CoV-1 and SARS-CoV-2. FXR2 paralog FMRP was previously shown to broadly bind along the entirety the 3′ UTR of the Zika virus (ZIKV) (75). However, while FMRP was suggested to act as a ZIKV restriction factor by blocking viral RNA translation, a significantly reduced ZIKV infection was observed upon knockdown of FXR2 (75).

#### Predicted FTO binding site is conserved in the 3′ UTR of SARS-CoV-1 and SARS-CoV-2

Altered expression levels of methyltransferase-like 3 (METTL3) and fat mass and obesity-associated protein (FTO) have been recently linked to viral replication (76). FTO is a demethylase (eraser) enzyme with enriched binding in the 3′ UTR of mRNAs in mammals (77). FTO has previously been suggested as a potential drug target against COVID-19 (78), as targeted knockdown has been shown to significantly decrease SARS-CoV-2 infection (76,78,79). Therefore, we investigated predicted binding of FTO to the 3′ UTR of SARS-CoV-2 and related viruses. Indeed, we observed that SARS-CoV-1, SARS-CoV-2 and MERS, as well as the less pathogenic viruses HCoV-HKU1 and HCoV-OC43 harbor at least one predicted FTO binding site in their 3′ UTR (Figure 5B). Further, Figure 5D shows that while SARS-CoV-1 and MERS harbor multiple shared predicted FTO binding sites along their 5′ UTR, SARS-CoV-2 only harbors one predicted FTO binding site at the 3′ end of its 5′ UTR which is exclusively shared with SARS-CoV-1.

#### Predicted TARDBP binding is newly acquired in the SARS-Cov-2 5′ UTR

We next focus on TARDBP (also known as TDP-43) (Figure 5F), which was predicted to bind the 5′ UTR of a SARS-CoV-2 mutant in a recent study (64). TARDBP, a host protein implicated in pre-mRNA alternative splicing, has been shown to play a role in viral replication and pathogenesis in the context of coxsackievirus B3 infection (80). In contrast to the findings of Mukherjee *et al.* (64), our model predicted a TARDBP binding site at the genomic range of 89-98 in the wild-type reference of SARS-CoV-2. Interestingly, in addition to observing a lack of predicted binding signal of TARDBP on the 5′ UTR of SARS-CoV-1 and MERS, we found a complete lack of predicted TARDBP binding to the 5’ UTR of any of the other investigated coronaviruses (Figure 5C). This suggests that 5′ UTR TARDBP binding potential may be newly acquired in SARS-CoV-2 and may affect its virulence.

### A functional catalog of human RBPs with predicted SARS-CoV-2 interaction

To understand the potential functional impact of predicted RBP binding sites on the SARS-CoV-2-mediated COVID-19 disease, we set out to interrogate the breadth of publicly available OMIC research, thereby gathering supportive evidences for our 88 RBPs models (Figure 6). We collected 97 data sets across 22 studies, covering experimentally determined and predicted viral RNA - host RBP interactions as well as multi-level (OMICS) data related to SARS-CoV-2 cell line infections, shedding light on viral entry, protein–protein interactions and host cell regulation (Methods). Studies which are closer to disease phenotypes, like CRISPR cell survival assays and COVID-19 patient data, were also included. In addition, we collected evidence of direct involvement of RBPs in SARS-CoV-2 infection, as reported in the SIGNOR database, a manually curated resource of pathways and genes involved in SARS-CoV-2 (81). Through data integration we, report evidence of binding or regulation for 85 out of 88 RBP models.

We found that a large fraction (64 out of 88, 72.4%) of RBPs were identified to directly bind SARS-CoV-2 RNA using affinity-purification methods (18,19) (labeled as *Experimentally supported binders*, Figure 6), supporting the predicted interactions of these RBPs with the viral RNA. Only 32 out of our 88 RBPs (36.8%) were common to RBPs reported by other predictive methods, namely catRAPID (49) or PRISMNet (50). However, these methods predict and report only RBPs binding the viral UTRs and the spike S gene. In addition, they do not provide single nucleotide resolution of the predicted binding sites, therefore the number and location of our predicted sites is not directly comparable to those studies. We thus complement the knowledge on predicted binding site locations over SARS-CoV-2 RNA with 56 RBPs uniquely reported by our framework, 37 of which are experimentally supported for viral RNA interactions. Our holistic comparison revealed that the majority of explored RBPs were previously reported to be part of host-pathogen PPI networks, cellular pathways which are altered during infection by either SARS-CoV-2, SARS-CoV-1 or CRISPR knock-out screenings, including 17 RBPs with no experimental evidence of direct binding to SARS-CoV-2 (labeled as *Infection relevant predicted binders*, Figure 6). This highlights the importance of RBPs in the infection process, immune response and viral replication. Although no RBP co-localized with loci associated to COVID-19 severe disease courses (GWAS) under genome-wide significance, we identified 44 (50.6%) RBPs with nominal significance. When considering the total of 2,730 coding genes co-localizing nominally associated loci, this represents a significant enrichment for RBPs (odds ratio of 7.8, Fisher test *P*-value < 2.2e−16), suggesting their importance in patient’s course. Finally, a small set of our predicted binding RBPs was shown to be supported only from CRISPR screens or found deregulated across COVID-19 patients, without experimental evidence in molecular networks (labeled as *Disease relevant predicted binders*, Figure 6). Taken together, the large overlap between our predicted RBP binders and the different resources considered confirms that hijacking host RBPs might be crucial to the infection life cycle of the virus.

## DISCUSSION

Strong evidence suggests that human RBPs are critical host factors for viral infection by SARS-CoV-2, yet experimental data on the exact binding sites of RBPs across the SARS-CoV-2 genome is still parse, due to the high costs associated with performing large-scale CLIP-seq experiments. To combat this knowledge gap, we constructed the first *in silico* human-virus RBP-RNA interaction map for SARS-CoV-2 using predictions from pysster (25) and DeepRiPe (26) models trained on a large cohort of eCLIP and PAR-CLIP datasets, respectively. The use of high-capacity CNN classifiers represents a significant improvement over previous computational studies performing motif scanning over the SARS-CoV-2 genome (40,82), as it enables the learning of more complex binding syntax and thus the successful prediction of binding sites for RBPs with no clear sequence motif. This is evident by the fact that we observed high performance for RBPs without annotations of binding motifs in literature. On the other hand, we recovered known binding motifs for several RBPs (including QKI, RBFOX2 and TARDBP) using gradient-based attribution methods. Together with stringent performance evaluation and conservative selection of high-quality models, these results suggest that our models predict binding sites with high accuracy on the basis of genuine RNA sequence sequence. In a recent study, the PRISMNet deep learning model was used to infer binding of 42 host RBPs to the SARS-CoV-2 genome (50). However, PRISMNet predictions were restricted to the 5’ and 3’ viral UTR regions and are rather large, with some spanning over hundreds of nucleotides, while RBP binding usually only occurs across short stretches of RNA *in vivo*. In contrast, our approach predicted single-nucleotide binding probabilities across the entire viral genome and may therefore yield a more accurate picture of the binding landscape of human RBPs to SARS-CoV-2.

Our study predicted binding sites for RBPs known to interact with SARS-CoV-2, as well as RBPs without existing experimental evidence to directly binding to the SARS-CoV-2 RNA. Further, the predicted binding map provides a rich resource for future functional studies, in particular for investigating the role of the SARS-CoV-2 protein–RNA interactome in context of the viral life cycle. For instance, binding site predictions may be used to accelerate the discovery of host RBPs that engage in both pro- and anti-viral functions by directly interacting with the viral RNA. Further, predictions may aid in the identification of functional sites on the viral RNA that can be therapeutically targeted by RNA drugs, such as anti-sense oligonucleotides, to interfere with host RBP binding. In addition to constructing a RBP binding map based on predicted binding sites on the SARS-CoV-2 reference sequence, we quantified the impact of sequence variants via *in silico* mutagenesis across 11 SARS-CoV-2 strains, including the alpha, delta and omicron viral strains.

Additionally, we performed systematic *in silico* mutagenesis of all positions in the SARS-CoV-2 genome, pinpointing mutations associated with particularly high impact, which could represent potential high-risk variants to monitor in the future. Through computation of feature importance scores on the reference and alternative sequence, our method revealed how sequence variants impact protein–RNA interactions. In previous studies, variants of concerns have been prioritized through their potential impact on the sequence of viral proteins, in particular the Spike protein. Our results complement these finding by giving insight into how strain-defining sequence mutations of variants of concern affect RNA regulatory elements, which may explain their impact on viral efficiency. With our comparative analysis of predicted RBP-RNA interactions across seven coronaviruses, we contribute towards the identification of RNA regulatory elements that are exclusive to SARS-CoV-2 and may therefore modulate its transmission and pathogenicity, compared to SARS-CoV-1, MERS and less pathogenic coronaviruses. Both the variants of concern and comparative analysis highlight predicted gain- or loss-of-binding events and therefore pinpoint RBPs which can be prioritized for further screening.

We integrated knowledge of RBPs with predicted binding sites on the SARS-CoV-2 RNA across other pathogens, host-viral protein–protein interactions, numerous studies collecting functional and phenotypic data, such as GWAS and CRISPR screens, as well as multi-omics COVID-19 patient data, in order to pinpoint RBPs with potential clinical significance.

Among RBPs with predicted binding sites on SARS-CoV-2 which are part of experimental protein–RNA interaction assays, we find several known regulators of viral processes. For example, we find the viral restriction factor hnRNPR (66), the IGF2BP1-3 RBPs, which are linked, through GWAS, to poor disease outcome (83), key regulators of SARS-CoV-2 infection CAPRIN1 and KHDRBS1 (84), the pro-viral factor pro-DDX3X (61) and the host factor NONO (66). Predicted binding of many RBPs was impacted by mutations in SARS-CoV-2 variants of concern, or showed changes when compared to other coronaviruses. For instance, TARDBP may be of particularly interest in the context of SARS-CoV-2 infection due to a unique predicted binding site in the virus 5’ UTR, compared to SARS-CoV-1, MERS and other coronaviruses.

Another RBP of interest is the Serine/arginine-rich splicing factor 7 (SRSF7), previously shown to interact with coronavirus RNA (85). It has been suggested that this spliceosome protein could be sequestrated by the viral genome, the later thus acting as a sponge through these putative binding sites, to alter host splicing processes. Among the high-impact mutations in the SRSF7 gene, position 23,604 (S protein gene) is found mutated across multiple strains, with different alternative nucleotides: a C>A transversion is found in alpha and mu variants, while a C>G transversion is found in delta and kappa variants. Both mutations are associated to a positive delta score, i.e. a predicted gain-of-binding. Besides SRSF7, the large number of predicted binding sites for splicing factors at the 5’ UTR of the SARS-CoV-2 (cluster 6, Figure 3C) and the significant enrichment of host and viral restriction RBPs in cluster 4 (Figure 3C), might suggest that these RBPs are likely to get sponged on the viral genome and by that modulate post-transcriptional regulatory networks in the host cell.

One other interesting RBP is represented by FXR2, paralog of FXR1 and FMR1 which are experimentally identified as direct binders of SARS-CoV-2 (Figure 6). Recent evidence suggests that FXR2 selectively interact with MERS viral proteins but not with viral proteins from SARS-CoV-1 and SARS-CoV-2 (28). While we find evidence of predicted FXR2 binding along the SARS-CoV-2 genome, this is in agreement with the results of our comparative analysis with other human coronaviruses, where we predict extensive binding of FXR2 along the 3′ UTR of SARS-CoV-1 and MERS, but depletion of FXR2 binding in the SARS-CoV-2 3′ UTR. Together with the evidence of genetic association of FXR2 to COVID-19 disease severity (86), our findings might suggest a role of FXR2 regarding the severity of the infection, although the physiological relevance of this RBPs remains to be experimentally assessed.

SARS-Cov-2 utilizes the endoplasmic reticulum (ER)-derived double membrane vesicles (DMVs) as replication centers. RNA viruses, included SARS-CoV-2, contains several instances of an RNA regulatory motif, called SECReTE motif (67) which facilitates localization to the ER and increases viral protein translation, as well as viral replication. Such motif is also found in some human mRNAs encoding for proteins involved in innate immunity and associated with epithelial layers targeted by SARS-CoV-2. This suggests that host and pathogen might compete for ER-associated RBPs. Indeed, we identified two clusters (7 and 8, Figure 3C) which harbor RBPs with binding sites significantly overlapping with SECReTE motifs. These include FUBP3, KHSRP and MATR3 (cluster 8), already identified previously as important host or restriction factors for other RNA virus infections (66). Interestingly, we linked MATR3 to several CRISPR studies showing that this factor is essential for SASR-CoV-2 replication, as well as to many nominal variants in all GWAS data (Figure 6). MATR3 physically interacts with G3BP1, another predicted RBP in this set which has been found to interact specifically with the SARS-CoV-2 nucleocapsid (N) protein, controls viral replication and localizes (together with MATR3) at stress granules where G3BP3 is taken away from its typical interactions partners, thereby impairing stress granule formation (87). The fact that G3BP3 binding is enriched in correspondence of the gene encoding for protein N (Figure 3C) might also suggest a direct regulation of this transcript by G3BP3 in a feedback loop manner.

The fat mass and obesity-associated protein (FTO) is an example of a RBP with predicted binding sites that has not been previously suggested to bind to the SARS-CoV-2 RNA. Besides the predicted binding pattern, FTO also presented numerous important gain- or loss-of-binding across many viral strains. Although there was no clear trend towards systematic loss-of-binding of FTO across the viral variants, we were able to point out multiple close-by mutations in the alpha variant that were predicted to be associated to a significant loss, around the position 28,280 (Figure 4B). Finally, the FTO protein was identified as key risk factor for obesity by other studies (Figure 6, GWAS, variant lowest *P*-value 0.0053), which is also a known risk for COVID-19 severity. A small set of RBPs showed predicted binding sites by our approach, while showing little to no experimental evidence across multiple functional studies. This includes the ELAVL2-4 factors (Figure 6). Interestingly, ELAVL2-4 RBPs, predicted from our analysis to be SECReTE motif-associated RBPs, were also found to be deregulated in COVID-19 patients (Figure 6). These RBPs might represent promising candidates whose molecular mechanisms can be further investigated experimentally. Our study provides a resource of computationally predicted binding sites of human RBPs to the SARS-CoV-2 RNA at high resolution - information that is currently not available through MS-based pull-down experiments. It is, however, important to point out some of the limitations of our approach. First, our method predicts the set of *potential* sites that may be bound by a protein of interest, rather than a specific configuration of sites occupied with binding proteins *in vivo*, with the later being, among other factors, highly cell-type specific. Further experiments are required to assess the role of those proteins (and their predicted binding sites) in a SARS-CoV-2 infection context. Second, as demonstrated in the comparison with CNBP eCLIP experimental data, our method misses some of the experimentally observed binding sites (Supplementary Figure S1). We believe that a certain fraction of false negative predictions correspond to viral-specific structures recognized by a host RBP, with a specific sequence composition which was probably never observed in human peaks and therefore not captured by a model trained on human data. It remains to be demonstrated whether future improvements to our current method, to incorporate knowledge of viral-specific sequences bound by host RBPs in the learning framework, could improve the method’s recall. All in all, our resource will help to prioritize RBPs for experimental investigation and direct future efforts towards promising candidates.

## CONCLUSION

Viruses depend on essential host factors at all stages of their infection cycle. One family of host factors, RNA-binding proteins (RBPs), are involved in multiple aspects of post-transcriptional regulation. While several RBPs have been associated with SARS-CoV-2, some of which may represent drug-able targets for anti-viral therapy, cost and time constraints render a comprehensive experimental profiling of human RBPs to the SARS-CoV-2 RNA at high spatial resolution infeasible. Mapping binding sites of human RBPs to the exact locations in the viral RNA is, however, crucial for gaining a mechanistic understanding of viral interaction with the host’s post-transcriptional machinery. We used the pysster and DeepRiPe deep learning frameworks together with data from CLIP-seq experiments to create an *in silico* binding map of human RBPs along the SARS-CoV-2 genome at single-nucleotide resolution. Predicted binding profiles of RBPs suggested that groups of RBPs exhibit similar binding patterns on the viral genome and that RBPs within these group may be functionally related, for example, by being associated to the SECReTE motif - a motif previously associated with efficient viral replication. We further utilized trained models to score the impact of strain-defining sequence variants across 11 SARS-CoV-2 strains and predicted several gain-or loss-of-binding events, some of which simultaneously impact the binding of multiple RBPs or are conserved in multiple viral strain. In addition, we quantified the impact of hypothetical variants by performing extensive *in silico* mutagenesis, thereby generating all possible point mutations across the SARS-CoV-2 genome. Finally, we predicted RBP-binding across 6 other human coronaviruses (including SARS-CoV-1 and MERS) and identified several conserved binding site predictions as well newly predicted binding sites in SARS-CoV-2. All generated results, including fully trained models, predicted binding sites across SARS-CoV-2 and other coronaviruses and variant impact scores are publicly available at *https://sc2rbpmap.helmholtz-muenchen.de/*. We believe that our results can help to give new insights into the role of RNA-binding proteins in context of SARS-CoV-2 infection and represent a rich resource for further research on how SARS-CoV-2 hijacks the host cell’s RNA regulatory machinery.

## DATA AVAILABILITY

Pre-trained models, together with scripts for training and prediction as well as binding site predictions and variant impact scores are available at https://github.com/mhorlacher/sc2rbpmap and https://doi.org/10.5281/zenodo.7547354. Graphical representations of predicted RBP binding sites on the SARS-CoV-2 genome and variant impact scores for variants of concern are available at https://sc2rbpmap.helmholtz-munich.de.

## Supplementary Material

lqad010_Supplemental_File
