# Supplementary material for: A computational map of the human-SARS-CoV-2 protein–RNA interactome predicted at single-nucleotide resolution
Source: NAR Genom Bioinform. 2023 Feb 20;5(1):lqad010. doi: 10.1093/nargab/lqad010 (PMC9940458; doi:10.1093/nargab/lqad010)
Supplement: lqad010_Supplemental_File [file lqad010_supplemental_file.pdf]

SUPPLEMENTARY TABLES

Supplementary Table 1: Overlap of pysster and DeepRiPe models with proteins from external sources

	pysster			DeepRiPe		Overlaps with proteins from external sources											
RBP	auROC	auPRC	medSCC	auROC	AP	[1]	[2]	[3]	[4]	[5]	[6]	[7]	[8]	[9]	[10]	[11]	SIGNOR*
AATF	0.92	0.66	0.1				X										
AGGF1	0.91	0.71	0.17									X			X[15]		
AGO1				0.79	0.32					X[4]							
AGO2				0.85	0.5												
AGO3				0.87	0.49												
AKAP8L	0.89	0.6	0.21				X					X					
AQR	0.93	0.7	0.22						X								
CAPRIN1				0.76	0.22					X[2,3,4]					X[14,16]	X[18]	Innate response to dsRNA, ER stress, Stress granules
CNBP	0.94	0.72	0.33					X		X[2,3,4]				X[8]			
CPSF1				0.77	0.23			X							X[15,16]		
CPSF6	0.89	0.61	0.18	0.79	0.26												
CPSF7				0.79	0.54								X[6]		X[15,16]	X[18]	
CSTF2	0.93	0.81	0.14	0.82	0.3											X[17,18]	
CSTF2T	0.92	0.6	0.19	0.84	0.66												
DDX3X	0.96	0.78	0.32							X[2,3]		X		X[10]	X[15]		
DDX59	0.89	0.67	0.16							X[4]							
DKC1	0.96	0.89	0.21									X			X[15,16]		
DND1				0.82	0.46												
EIF4G2	0.95	0.78	0.31					X		X[4]					X[15,16]		
ELAVL1				0.9	0.73			X			X		X[6,7]	X[8]	X[15,16]		
ELAVL2				0.93	0.61												
ELAVL3				0.94	0.72												
ELAVL4				0.93	0.58												
EWSR1	0.93	0.62	0.22	0.85	0.2				X[1]					X[9]	X[12,16]	X[18]	
FAM120A	0.92	0.62	0.24								X			X[8]	X[15,16]	X[17,18]	
FIP1L1				0.8	0.3					X[4]					X[15,16]		
FKBP4	0.93	0.65	0.18										X[7]		X[14,16]		Virus entry
FMR1	0.94	0.67	0.18							X[4]		X		X[10]			
FTO	0.92	0.63	0.27														
FUBP3	0.95	0.8	0.14							X[2,3]				X[8]			
FXR1	0.92	0.6	0.26	0.86	0.26	X								X[8]			
FXR2	0.94	0.67	0.23	0.8	0.18	X								X[10]			
G3BP1	0.93	0.64	0.31			X	X			X[2,3,4]				X[8]	X[11,15,16]		Innate response to dsRNA, Inflammation, ER stress, Cytokine Storm
GPKOW	0.92	0.71	0.16					X									
GRSF1	0.93	0.71	0.18						X	X[4]							
GTF2F1	0.94	0.71	0.29							X[4]					X[15]	X[17]	
HNRNPA1	0.94	0.74	0.11							X[2,3]	X				X[15]	X[17,18]	
HNRNPC	0.97	0.83	0.15								X		X[6,7]		X[15,16]	X[18]	
HNRNPD				0.94	0.47								X[7]	X[10]	X[15,16]		
HNRNPK	0.98	0.87	0.3								X		X[6,7]		X[15,16]	X[17]	
HNRNPL	0.97	0.86	0.31							X[2,3]	X		X[6]	X[10]			
HNRNPM	0.95	0.74	0.22					X			X	X	X[7]	X[9]			
IGF2BP1	0.91	0.66	0.12	0.83	0.19					X[2,3]	X	X	X[6]		X[14,16]	X[17]	
IGF2BP2	0.91	0.65	0.13	0.84	0.29					X[2]		X		X[8]		X[17]	
IGF2BP3	0.88	0.56	0.08	0.84	0.42						X	X		X[8]	X[14]	X[17,18]	
ILF3	0.93	0.74	0.15							X[4]			X[7]	X[9]	X[15,16]	X[17]	
KHDRBS1	0.97	0.86	0.18								X			X[9]	X[15,16]	X[17,18]	Innate response to dsRNA, Inflammation, MAPK activation, Stress granules, Cytokine Storm
KHSRP	0.9	0.65	0.15								X	X			X[15,16]	X[17,18]	Apoptosis, Fibrosis, Innate response to dsRNA, Virus entry, Inflammation, ER stress, MAPK activation, Stress granules, Cytokine Storm
L1RE1				0.96	0.59		X				X						
MATR3	0.94	0.7	0.23					X		X[4]	X			X[8]	X[15,16]		
MBNL1				0.98	0.94										X[16]		
NCBP2	0.93	0.71	0.24														
NIP7	0.92	0.69	0.15										X[7]				
NONO	0.92	0.6	0.15	0.93	0.38			X					X[7]	X[8,9]	X[15,16]	X[17]	

NOP56				0.92	0.69										X[15,16]	X[17,18]	
NOP58				0.93	0.68					X[4]							
ORF1																	
PCBP1	0.93	0.67	0.16											X[10]	X[15,16]		
PCBP2	0.96	0.79	0.3					X		X[2,3]					X[15]		Fibrosis, Innate response to dsRNA, Virus entry, Inflammation, ER stress, MAPK activation, Stress granules, Cytokine Storm
PRPF8	0.95	0.74	0.33												X[14]		
PTBP1	0.94	0.8	0.37								X		X[6,7]	X[8]	X[15,16]	X[17,18]	
PUM2	0.95	0.8	0.16	0.95	0.72									X[10]	X[15,16]		
QKI	0.97	0.87	0.31	0.97	0.64											X[17,18]	
RBFOX2	0.96	0.8	0.24												X[15,16]		
RBM20				0.91	0.59												
RBM22	0.91	0.72	0.2													X[17,18]	
RBPMS				0.97	0.78										X[13]		
RPS3	0.94	0.63	0.28							X[2,3,4]				X[10]	X[12,14,15,16]		
SAFB2	0.93	0.7	0.11											X[9]	X[15]	X[18]	
SF3A3	0.96	0.84	0.23														
SF3B4	0.98	0.88	0.26			X		X									
SND1	0.95	0.75	0.21							X[2,3,4]			X[5]	X[8]	X[15]	X[17,18]	
SRRM4				0.8	0.31												
SRSF1	0.94	0.7	0.28								X		X[6,7]		X[15,16]		
SRSF7	0.92	0.69	0.17					X		X[4]	X				X[16]		
SRSF9	0.92	0.65	0.18												X[14,15,16]		
SUGP2	0.9	0.63	0.15							X[4]							
TAF15	0.93	0.7	0.17	0.88	0.28										X[15]		
TARDBP	0.98	0.92	0.28	0.95	0.73			X			X					X[18]	Apoptosis, Fibrosis, Innate response to dsRNA, Virus entry, Inflammation, ER stress, MAPK activation, Stress granules, Cytokine Storm
TIAL1	0.95	0.8	0.13							X[4]				X[10]		X[17,18]	
TRA2A	0.96	0.8	0.29												X[14,15,16]		
U2AF2	0.95	0.77	0.15										X[6,7]			X[17,18]	
XRN2	0.93	0.64	0.18												X[15]		
YBX3	0.92	0.71	0.1							X[2]				X[8]			
ZC3H7B				0.87	0.37												
ZFP36				0.93	0.46					X[4]					X[15,16]		
ZNF800	0.93	0.62	0.25							X[4]		X			X[15]		
ZRANB2	0.9	0.64	0.1							X[4]							

[1] also included in the PPI network

[2] SARS-CoV-2 RNA interacting proteins

[3] proteins included in the PPI network (network based on STRING v.11 interactions between human proteins in the expanded SARS-CoV-2 RNA interactome)

[4] differentially expressed proteins (SARS-CoV-2 infected and uninfected Huh7 cells)

[5] proteins that were reduced during SARS-CoV-2 infection

[6] proteins that increased during SARS-CoV-2 infection

[7] additional potential inhibitors of SARS-CoV-2 replication

[8] statistically significant interactors enriched in both probe I and probe II experiment

[9] statistically significant interactors enriched in only probe I experiment

[10] statistically significant interactors enriched in only probe II experiment

[11] proteins included in virus-host PPI network of SARS-CoV-2 in A549 cells

[12] transcripts that significantly change upon SARS-CoV-2 infection

[13] proteins that significantly change upon SARS-CoV-2 infection

[14] ubiquitination site significantly changes upon SARS-CoV-2 infection

[15] phosphorylation site significantly changes upon SARS-CoV-2 infection

[16] included in results of enrichment analysis

[17] RBPs predicted to bind the 5'UTR of SARS-CoV-2

[18] RBPs predicted to bind the 3'UTR of SARS-CoV-2

[*] empirical p-value < 0.05

Color	# overlaps
	0
	1
	2
	3
	4
	5
	6

Supplementary Table 2: Correlation coefficient from genome-wide SARS-CoV-2 prediction scores between pyssster models and pre-trained DeepRiPe models trained on matched ENCODE eCLIP experiments. The average correlation across RBPs was 0.32, indicative of highly correlated predictions between the two tools. This is especially true for RBPs with known sequence motifs, such as QKI (0.71), RBFOX2 (0.56) and PUM2 (0.59).

	RBP_CELL	PCC	RBP_CELL	PCC	RBP_CELL	PCC
1	QKI_HepG2	0.71	KHDRBS1_K562	0.44	AQR_HepG2	0.19
2	KHSRP_HepG2	0.60	FXR1_K562	0.42	NONO_K562	0.17
3	PUM2_K562	0.59	SRSF1_HepG2	0.41	NCBP2_HepG2	0.14
4	ZNF800_HepG2	0.58	SF3A3_HepG2	0.40	EWSR1_K562	0.13
5	HNRNPL_HepG2	0.57	U2AF2_HepG2	0.37	FAM120A_HepG2	0.13
6	RBFOX2_HepG2	0.56	SUGP2_HepG2	0.36	PCBP2_HepG2	0.13
7	FXR2_HepG2	0.55	SF3B4_HepG2	0.36	ILF3_HepG2	0.13
8	FUBP3_HepG2	0.55	SAFB2_K562	0.35	AGGF1_HepG2	0.10
9	RPS3_HepG2	0.54	TIAL1_HepG2	0.33	XRN2_HepG2	0.09
10	TARDBP_K562	0.54	HNRNPC_HepG2	0.33	GTF2F1_HepG2	0.09
11	HNRNPM_HepG2	0.53	PTBP1_HepG2	0.33	DKC1_HepG2	0.07
12	MATR3_HepG2	0.53	RBM22_HepG2	0.32	GRSF1_HepG2	0.06
13	CSTF2_HepG2	0.51	FMR1_K562	0.32	CSTF2T_HepG2	0.03
14	G3BP1_HepG2	0.51	PRPF8_HepG2	0.31	HNRNPK_HepG2	0.02
15	SND1_HepG2	0.49	IGF2BP2_K562	0.28	DDX59_HepG2	0.01
16	DDX3X_HepG2	0.46	GPKOW_K562	0.27	EIF4G2_K562	-0.02
17	YBX3_HepG2	0.45	AATF_K562	0.22	AKAP8L_K562	-0.04
18	IGF2BP1_HepG2	0.45	FKBP4_HepG2	0.19		

Supplementary Table 3: Comparison of high quality pysster and DeepRiPe models

**RBP**	**cor of pred score**	**cor of p-value**	**# common binding sites**
TARDBP	0.640832	0.40309	6
CSTF2	0.459011	0.21621	6
IGF2BP1	0.387823	0.39983	9
PUM2	0.383309	0.35263	10
CSTF2T	0.331395	0.22239	4
QKI	0.279760	0.14371	5
IGF2BP2	0.171838	0.21092	7
IGF2BP3	0.073798	0.05951	5
CPSF6	0.153344	0.26078	2
FXR1	0.012354	0.14136	8
FXR2	0.080191	0.19433	5
EWSR1	0.009787	0.06610	0

Supplementary Table 4: Tested significance on pysster ENCODE models, DeepRiPe models and pysster model trained on CNBP

	Model	RBP	p-value	FDR	Num. binding sites	Mean, H_0	Std., H_0	Model	RBP	p-value	FDR	Num. binding sites	Mean, H_0	Std., H_0
1	AATF_K562	AATF	0.92	1.00	22	30.18	6.45	IGF2BP3_HEK293	IGF2BP3	0.64	0.99	23	24.09	4.71
2	**AGGF1_HepG2**	**AGGF1**	**0.01**	**0.03**	**35**	**9.89**	**3.36**	ILF3_HepG2	ILF3	0.59	0.97	9	9.84	4.42
3	AGO1_HEK293	AGO1	0.38	0.69	22	20.27	4.63	KHDRBS1_K562	KHDRBS1	0.99	1.00	10	17.85	4.68
4	AGO2_HEK293	AGO2	0.14	0.30	18	12.81	4.19	KHSRP_HepG2	KHSRP	0.79	1.00	16	19.08	4.54
5	AGO3_HEK293	AGO3	0.31	0.57	20	17.63	4.48	L1RE1_HEK293	L1RE1	0.70	1.00	13	15.60	4.69
6	AKAP8L_K562	AKAP8L	0.04	0.10	10	4.47	2.12	**MATR3_HepG2**	**MATR3**	**0.03**	**0.08**	**16**	**7.54**	**3.14**
7	AQR_HepG2	AQR	0.07	0.16	30	19.95	5.84	**MBNL1_HEK293**	**MBNL1**	**0.01**	**0.03**	**19**	**10.45**	**3.51**
8	CAPRIN1_HEK293	CAPRIN1	0.96	1.00	19	27.12	4.86	NCBP2_HepG2	NCBP2	0.71	1.00	9	10.41	3.28
9	**CNBP_HuH7**	**CNBP**	**0.01**	**0.03**	**8**	**1.60**	**1.15**	**NIP7_HepG2**	**NIP7**	**0.01**	**0.03**	**43**	**24.02**	**5.33**
10	**CPSF1_HEK293**	**CPSF1**	**0.02**	**0.06**	**28**	**18.25**	**4.50**	NONO_K562	NONO	1.00	1.00	8	23.07	6.00
11	**CPSF6_K562**	**CPSF6**	**0.01**	**0.03**	**45**	**22.81**	**5.11**	NOP56_HEK293	NOP56	0.51	0.91	12	11.65	2.95
12	CPSF7_HEK293	CPSF7	0.26	0.51	27	23.76	4.21	**NOP58_HEK293**	**NOP58**	**0.01**	**0.03**	**26**	**11.59**	**3.35**
13	CSTF2_HepG2	CSTF2	1.00	1.00	10	16.84	4.14	ORF1_HEK293	ORF1	0.39	0.69	14	12.63	3.50
14	CSTF2T_HepG2	CSTF2T	0.93	1.00	4	7.56	2.89	**PCBP1_HepG2**	**PCBP1**	**0.01**	**0.03**	**8**	**0.73**	**0.94**
15	**DDX3X_HepG2**	**DDX3X**	**0.01**	**0.03**	**17**	**6.77**	**2.93**	PCBP2_HepG2	PCBP2	1.00	1.00	0	0.78	0.93
16	DDX59_HepG2	DDX59	1.00	1.00	0	0.21	0.46	PRPF8_HepG2	PRPF8	0.18	0.37	19	14.69	3.83
17	**DKC1_HepG2**	**DKC1**	**0.01**	**0.03**	**16**	**6.58**	**2.79**	**PTBP1_HepG2**	**PTBP1**	**0.02**	**0.06**	**3**	**0.62**	**0.72**
18	DND1_HEK293	DND1	0.60	0.97	14	14.33	3.86	**PUM2_K562**	**PUM2**	**0.03**	**0.08**	**29**	**15.84**	**4.91**
19	EIF4G2_K562	EIF4G2	0.10	0.22	5	2.27	1.54	QKI_HepG2	QKI	0.28	0.54	15	11.78	4.09
20	ELAVL1_HEK293	ELAVL1	0.96	1.00	7	13.66	3.71	RBFOX2_HepG2	RBFOX2	0.94	1.00	8	13.74	4.07
21	ELAVL2_HEK293	ELAVL2	0.57	0.97	18	18.56	4.12	**RBM20_HEK293**	**RBM20**	**0.01**	**0.03**	**30**	**17.65**	**4.46**
22	ELAVL3_HEK293	ELAVL3	0.59	0.97	15	15.32	3.89	**RBM22_HepG2**	**RBM22**	**0.01**	**0.03**	**45**	**19.18**	**4.41**
23	ELAVL4_HEK293	ELAVL4	0.68	1.00	14	15.51	3.92	RBPMS_HEK293	RBPMS	0.05	0.12	19	11.29	3.77
24	EWSR1_K562	EWSR1	1.00	1.00	0	0.43	0.64	**RPS3_HepG2**	**RPS3**	**0.01**	**0.03**	**57**	**20.43**	**5.72**
25	FAM120A_HepG2	FAM120A	0.94	1.00	2	4.55	2.28	**SAFB2_K562**	**SAFB2**	**0.01**	**0.03**	**44**	**21.28**	**4.96**
26	FIP1L1_HEK293	FIP1L1	0.79	1.00	15	17.95	4.69	SF3A3_HepG2	SF3A3	0.04	0.10	34	23.40	4.81
27	FKBP4_HepG2	FKBP4	1.00	1.00	0	0.01	0.10	SF3B4_HepG2	SF3B4	0.04	0.10	30	21.97	4.19
28	**FMR1_K562**	**FMR1**	**0.01**	**0.03**	**59**	**22.57**	**4.74**	**SND1_HepG2**	**SND1**	**0.01**	**0.03**	**82**	**24.94**	**5.66**
29	**FTO_HepG2**	**FTO**	**0.01**	**0.03**	**58**	**19.87**	**5.09**	SRRM4_HEK293	SRRM4	1.00	1.00	17	28.25	4.87
30	FUBP3_HepG2	FUBP3	0.66	1.00	20	20.99	4.75	**SRSF1_HepG2**	**SRSF1**	**0.01**	**0.03**	**22**	**11.33**	**3.09**
31	**FXR1_K562**	**FXR1**	**0.01**	**0.03**	**106**	**19.28**	**5.27**	SRSF7_HepG2	SRSF7	0.10	0.22	38	30.24	5.50
32	**FXR2_HepG2**	**FXR2**	**0.01**	**0.03**	**70**	**23.01**	**5.33**	**SRSF9_HepG2**	**SRSF9**	**0.01**	**0.03**	**28**	**13.45**	**3.13**
33	**G3BP1_HepG2**	**G3BP1**	**0.01**	**0.03**	**83**	**20.40**	**5.47**	SUGP2_HepG2	SUGP2	0.20	0.41	24	19.73	5.07
34	GPKOW_K562	GPKOW	0.07	0.16	28	18.75	5.23	TAF15_HepG2	TAF15	1.00	1.00	5	18.53	3.95
35	GRSF1_HepG2	GRSF1	0.88	1.00	7	9.97	3.27	TARDBP_K562	TARDBP	0.98	1.00	12	18.55	4.21
36	GTF2F1_HepG2	GTF2F1	0.79	1.00	1	1.49	1.26	TIAL1_HepG2	TIAL1	0.71	1.00	27	30.93	5.91
37	HNRNPA1_HepG2	HNRNPA1	1.00	1.00	16	28.20	5.29	**TRA2A_HepG2**	**TRA2A**	**0.01**	**0.03**	**47**	**19.39**	**4.87**
38	HNRNPC_HepG2	HNRNPC	1.00	1.00	14	26.67	5.33	U2AF2_HepG2	U2AF2	0.10	0.22	31	24.17	4.95
39	HNRNPD_HEK293	HNRNPD	0.84	1.00	12	15.30	4.04	XRN2_HepG2	XRN2	1.00	1.00	2	8.79	2.79
40	HNRNPK_HepG2	HNRNPK	0.64	0.99	1	1.36	1.31	**YBX3_HepG2**	**YBX3**	**0.01**	**0.03**	**60**	**25.24**	**6.67**
41	HNRNPL_HepG2	HNRNPL	0.55	0.96	9	8.76	3.13	ZC3H7B_HEK293	ZC3H7B	0.29	0.55	20	17.41	4.50
42	HNRNPM_HepG2	HNRNPM	1.00	1.00	12	25.99	5.66	ZFP36_HEK293	ZFP36	1.00	1.00	7	15.63	4.46
43	**IGF2BP1_HepG2**	**IGF2BP1**	**0.01**	**0.03**	**50**	**21.93**	**5.75**	**ZNF800_HepG2**	**ZNF800**	**0.01**	**0.03**	**39**	**12.33**	**4.17**
44	**IGF2BP2_K562**	**IGF2BP2**	**0.01**	**0.03**	**50**	**29.53**	**5.25**	**ZRANB2_K562**	**ZRANB2**	**0.01**	**0.03**	**34**	**21.58**	**4.91**

Supplementary Table 5: Overlap of pysster and DeepRiPe binding sites with SECReTE motif

RBP	Num. overlaps	Num. binding sites	Ratio	Model	Binding sites
FUBP3	7	20	0.350	pysster	[11040,11049], [11068,11118], [14155,14165], [14383,14391], [21920,21928], [26299,26317], [26336,26344]
PTBP1	1	3	0.333	pysster	[9500,9584]
KHSRP	5	16	0.312	pysster	[8596,8635], [9496,9530], [11287,11326], [14142,14172], [26293,26351]
SUGP2	7	24	0.292	pysster	[9622,9652], [11091,11116], [11191,11231], [11278,11368], [11626,11663], [21580,21596], [27831,27848]
ELAVL4	4	14	0.286	DeepRiPe	[8622,8624], [11028,11065], [27677,27685], [27802,27824]
ZFP36	2	7	0.286	DeepRiPe	[8595,8613], [21928,21932]
ELAVL2	5	18	0.278	DeepRiPe	[8612,8624], [11029,11067], [21563,21565], [21911,21942], [27795,27827]
ELAVL3	4	15	0.267	DeepRiPe	[9523,9527], [11041,11065], [21920,21933], [27800,27826]
MBNL1	5	19	0.263	DeepRiPe	[11645,11657], [15492,15524], [26288,26341], [27164,27199], [27792,27853]
CSTF2T	1	4	0.250	pysster	[8598,8618]
MATR3	4	16	0.250	pysster	[9511,9539], [11182,11313], [11614,11658], [26303,26324]
AGO2	4	18	0.222	DeepRiPe	[8589,8631], [15528,15582], [24091,24134], [28722,28730]
NCBP2	2	9	0.222	pysster	[12026,12027], [14770,14772]
ZNF800	8	39	0.205	pysster	[1659,1701], [3826,4059], [12025,12234], [14769,14774], [14800,14882], [15529,15533], [28692,28764], [28911,28957]
CSTF2	2	10	0.200	pysster	[8591,8633], [13883,13912]
QKI	3	15	0.200	pysster	[9521,9533], [11326,11347], [14167,14178]
DDX3X	3	17	0.176	pysster	[12026,12030], [14769,14772], [28861,28949]
TARDBP	2	12	0.167	pysster	[11117,11123], [11218,11388]
G3BP1	12	83	0.145	pysster	[818,841], [1651,1731], [3787,4196], [8168,8346], [12026,12376], [14396,14397], [14769,14824], [14836,14908], [15519,15531], [16524,16810], [28386,28753], [28913,28974]
GRSF1	1	7	0.143	pysster	[28891,28903]
HNRNPC	2	14	0.143	pysster	[9787,9789], [11034,11044]
CPSF6	6	45	0.133	pysster	[1707,1715], [3808,4054], [4090,4098], [11182,11214], [21938,21969], [26333,26352]
FIP1L1	2	15	0.133	DeepRiPe	[13900,13935], [21935,21971]
SF3B4	4	30	0.133	pysster	[11959,12000], [16553,16561], [21527,21529], [24083,24101]
U2AF2	4	31	0.129	pysster	[9773,9777], [12004,12023], [14379,14392], [27785,27839]
CNBP	1	8	0.125	pysster	[28866,28916]
DKC1	2	16	0.125	pysster	[779,889], [14769,14801]
NONO	1	8	0.125	pysster	[11204,11222]
NIP7	5	43	0.116	pysster	[806,834], [13880,13945], [14763,14797], [16536,16546], [25236,25254]
CPSF1	3	28	0.107	DeepRiPe	[827,841], [23278,23312], [28914,28946]
GPKOW	3	28	0.107	pysster	[11982,12026], [21575,21579], [26302,26310]
SRSF9	3	28	0.107	pysster	[821,834], [12026,12028], [28850,28915]
FXR1	11	106	0.104	pysster	[639,928], [1631,1703], [1715,1723], [3963,4107], [8619,8627], [13907,13913], [14771,14876], [15506,15549], [16543,16555], [23231,23266], [27401,27417]
AKAP8L	1	10	0.100	pysster	[14786,14788]
AATF	2	22	0.091	pysster	[5698,5702], [15525,15568]
ZRANB2	3	34	0.088	pysster	[11655,11661], [21574,21586], [26284,26330]
AGGF1	3	35	0.086	pysster	[4031,4057], [12024,12088], [14766,14776]
FMR1	5	59	0.085	pysster	[750,839], [858,910], [14879,14891], [23268,23276], [28859,28908]
HNRNPD	1	12	0.083	DeepRiPe	[9492,9522]
HNRNPM	1	12	0.083	pysster	[11345,11384]
NOP56	1	12	0.083	DeepRiPe	[814,821]

SRSF7	3	38	0.079	pyssster	[820,838], [14771,14811], [23253,23280]
CPSF7	2	27	0.074	DeepRiPe	[14849,14875], [21924,21929]
FXR2	5	70	0.071	pyssster	[3776,4047], [4059,4063], [12024,12268], [13905,13957], [14770,14775]
ORF1	1	14	0.071	DeepRiPe	[28693,28703]
FTO	4	58	0.069	pyssster	[12021,12131], [14795,14809], [23258,23266], [28679,28778]
RBM22	3	45	0.067	pyssster	[4029,4057], [26286,26336], [28834,28935]
YBX3	4	60	0.067	pyssster	[705,870], [14767,14855], [14891,14919], [23295,23327]
SF3A3	2	34	0.059	pyssster	[11965,12009], [16529,16601]
SRRM4	1	17	0.059	DeepRiPe	[26278,26328]
PRPF8	1	19	0.053	pyssster	[27150,27193]
RPS3	3	57	0.053	pyssster	[730,941], [14769,14776], [28568,28740]
AGO3	1	20	0.050	DeepRiPe	[28710,28752]
SND1	4	82	0.049	pyssster	[750,852], [14771,14772], [23266,23287], [28697,28720]
AGO1	1	22	0.045	DeepRiPe	[28712,28771]
SAFB2	2	44	0.045	pyssster	[1688,1710], [4042,4062]
SRSF1	1	22	0.045	pyssster	[12024,12028]
TRA2A	2	47	0.043	pyssster	[12025,12055], [14766,14774]
TIAL1	1	27	0.037	pyssster	[26271,26318]
PUM2	1	29	0.034	pyssster	[27141,27165]
AQR	1	30	0.033	pyssster	[11957,12027]
RBM20	1	30	0.033	DeepRiPe	[28728,28736]
IGF2BP1	1	50	0.020	pyssster	[28657,28722]
CAPRIN1	0	19	0.000	DeepRiPe	
DDX59	0	0	0.000	pyssster	
DND1	0	14	0.000	DeepRiPe	
EIF4G2	0	5	0.000	pyssster	
ELAVL1	0	7	0.000	DeepRiPe	
EWSR1	0	0	0.000	pyssster	
FAM120A	0	2	0.000	pyssster	
FKBP4	0	0	0.000	pyssster	
GTF2F1	0	1	0.000	pyssster	
HNRNPA1	0	16	0.000	pyssster	
HNRNPK	0	1	0.000	pyssster	
HNRNPL	0	9	0.000	pyssster	
IGF2BP2	0	50	0.000	pyssster	
IGF2BP3	0	23	0.000	DeepRiPe	
ILF3	0	9	0.000	pyssster	
KHDRBS1	0	10	0.000	pyssster	
L1RE1	0	13	0.000	DeepRiPe	
NOP58	0	26	0.000	DeepRiPe	
PCBP1	0	8	0.000	pyssster	
PCBP2	0	0	0.000	pyssster	
RBFOX2	0	8	0.000	pyssster	
RBPMS	0	19	0.000	DeepRiPe	
TAF15	0	5	0.000	pyssster	
XRN2	0	2	0.000	pyssster	
ZC3H7B	0	20	0.000	DeepRiPe	

SUPPLEMENTARY FIGURES

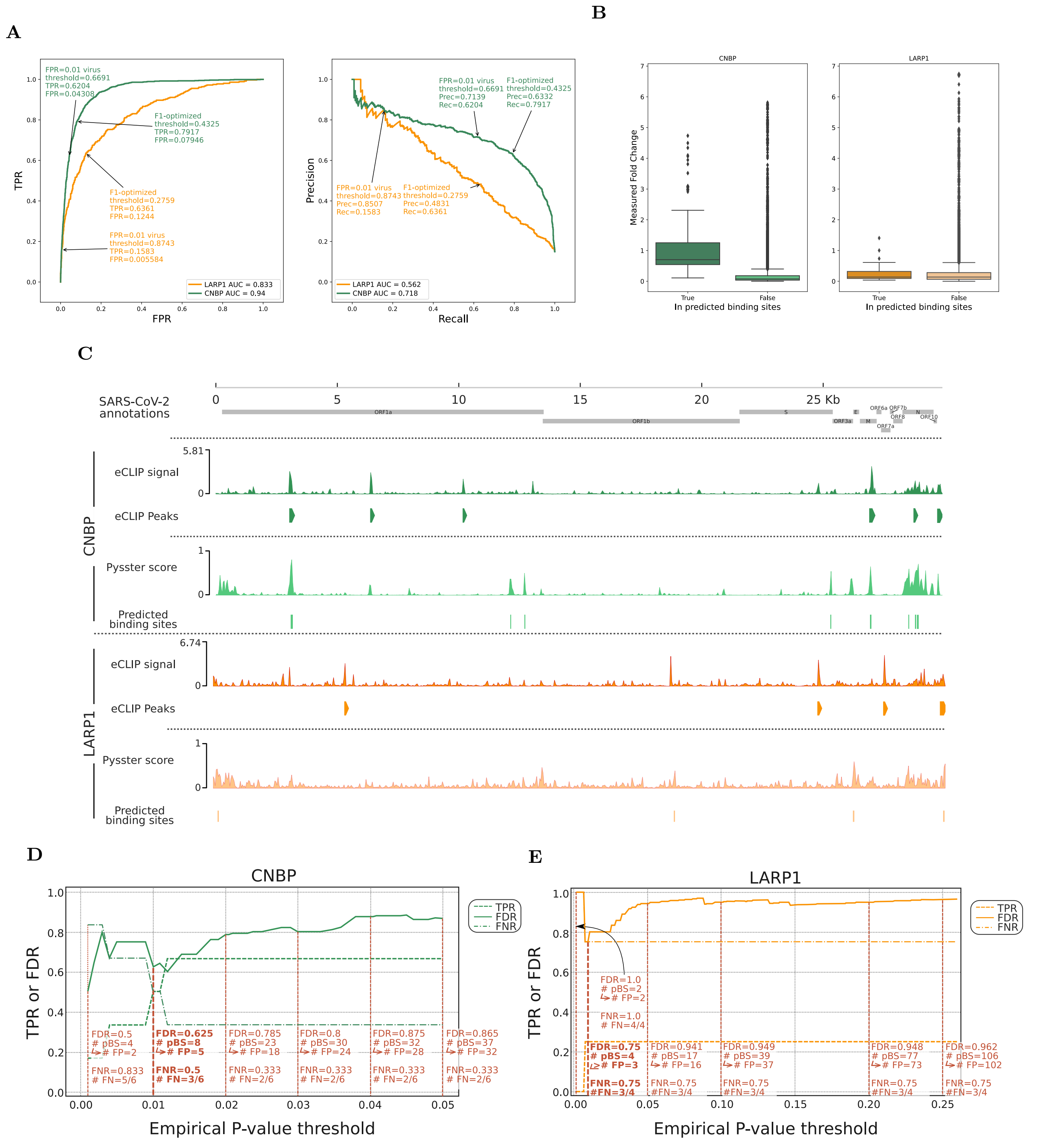

Supplementary Figure 1: Evaluation of additional models for LARP1 and CNBP. **a.** ROC and PRC from the train/test evaluation. For each model are annotated two score thresholds : the F1-optimized one, and the one equivalent to the empirical P-value threshold of 0.01 (False Positive Rate from the 10,000 sequences with viral dinucleotide frequences). Note: only the CNBP model passes the Area Under PRC threshold set for high confidence models. **b.** Boxplot of Fold Change values measured by eCLIP on the SARS-CoV-2 genome, comparing positions within predicted binding sites against other positions. **(**c.) Genome Browser view of the eCLIP signal (continuous Fold Change values and narrow peaks) and predictions from LARP1 and CNBP pysster models (continuous prediction scores and predicted binding sites). **d.** and **e.** Impact of the empirical P-value threshold on the False Discovery Rate (proportion of predicted Binding Sites not overlapping narrow peaks), True Positive Rate (proportion of narrow peaks overlapped by predicted Binding Sites), and False Negative Rate (1 - TPR) for CNBP (d.) and LARP1 (e.). The threshold range goes from the smallest value leading to at least one predicted binding site, to the empirical P-value equivalent to the F1-optimized score threshold. Various thresholds are annotated, with bold annotation for the chosen empirical P-value threshold of 0.01.

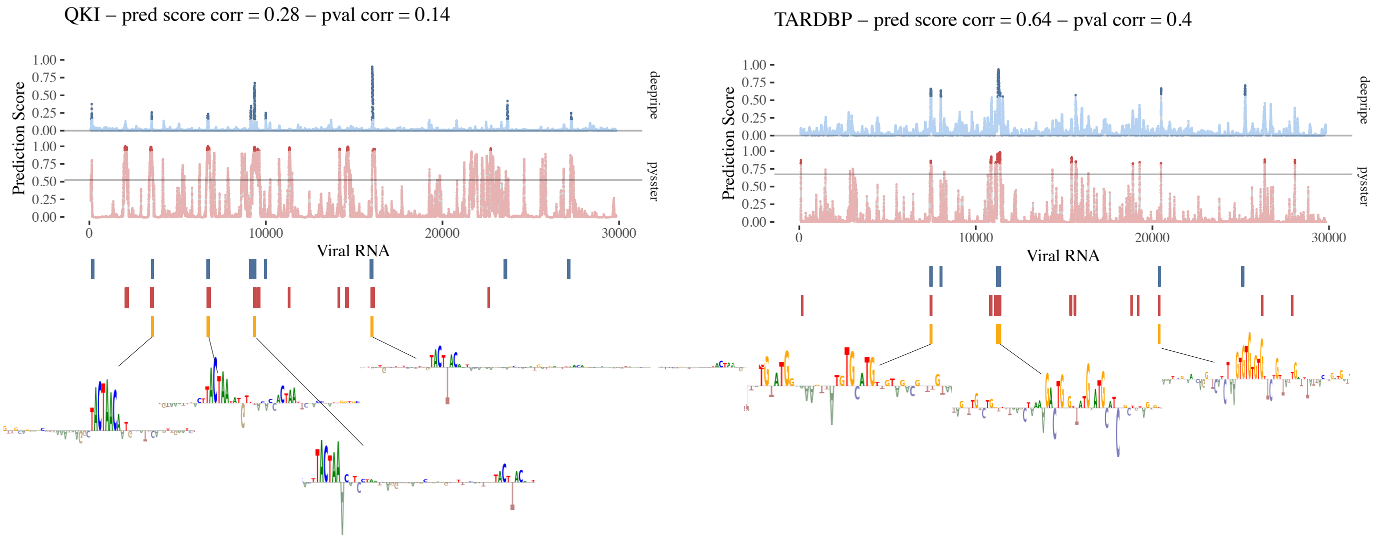

Supplementary Figure 2: RBP binding pattern on the SARS-CoV-2 genome between the two methods, pysster and DeepRiPE. Comparison of single-nucleotide probability scores of binding for two RBPs, QKI (left panel) and TARDBP (right panel). Significant binding sites, commonly predicted by both methods are shown underneath the probability plots together with their corresponding learnt motifs from the attribution maps. Prediction score correlation and p-value correlation given in the header.

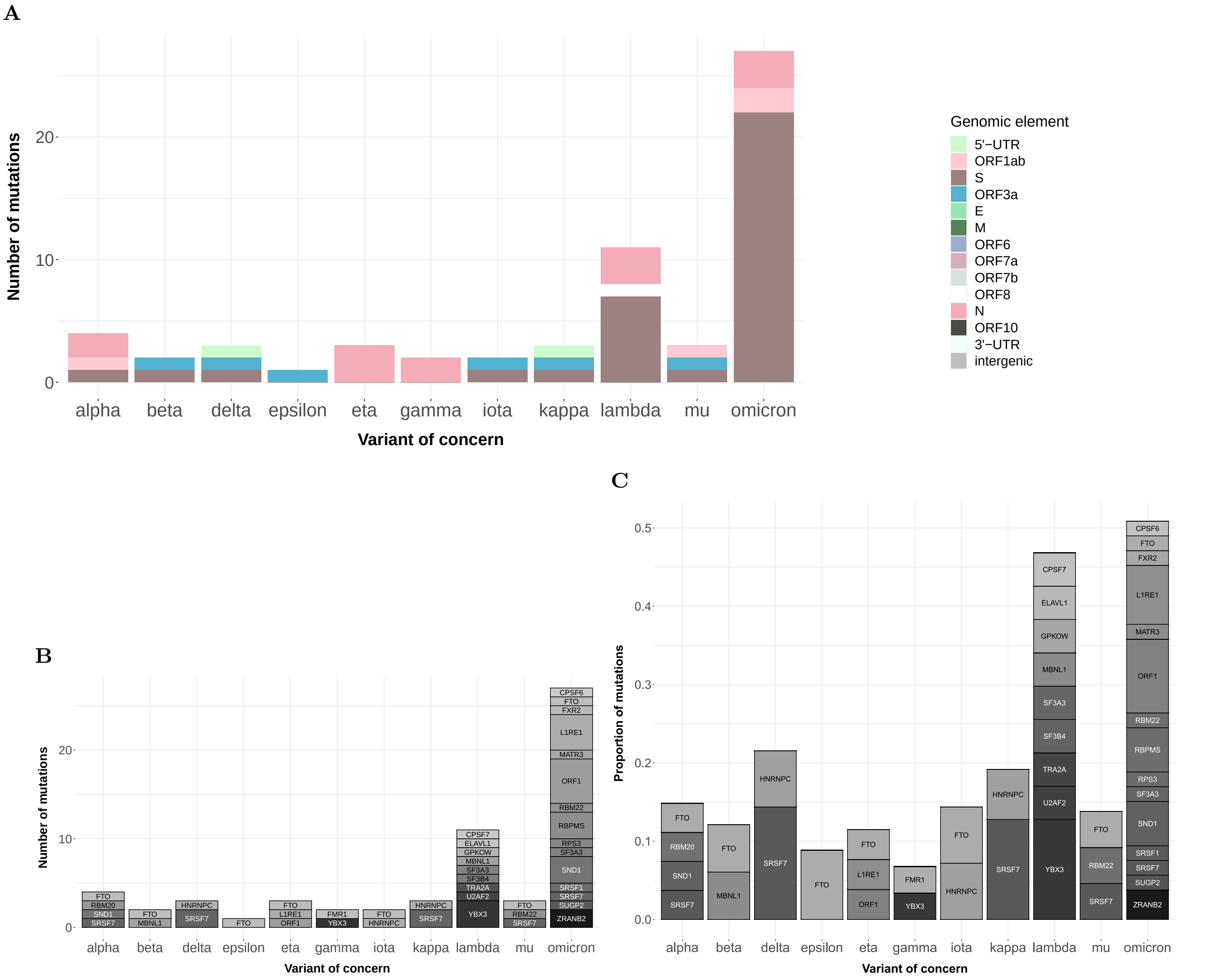

Supplementary Figure 3: Impact of mutations from variants of concern on predicted binding sites. **a**. Accumulation of predicted high-impact mutations in viral components for each variant of concern. The subset of high-impact mutations here corresponds to the one represented in Figure 4A, i.e. the top 20% of predicted binding-impacting mutations. **b**. Accumulation of impacted RBP sites for each variant of concern. The same subset as in (a) was used here. **c**. Same as in (b), but reporting the proportion of high-impacting mutations per RBP over the total of mutations for a variant of concern, rather than their absolute number.

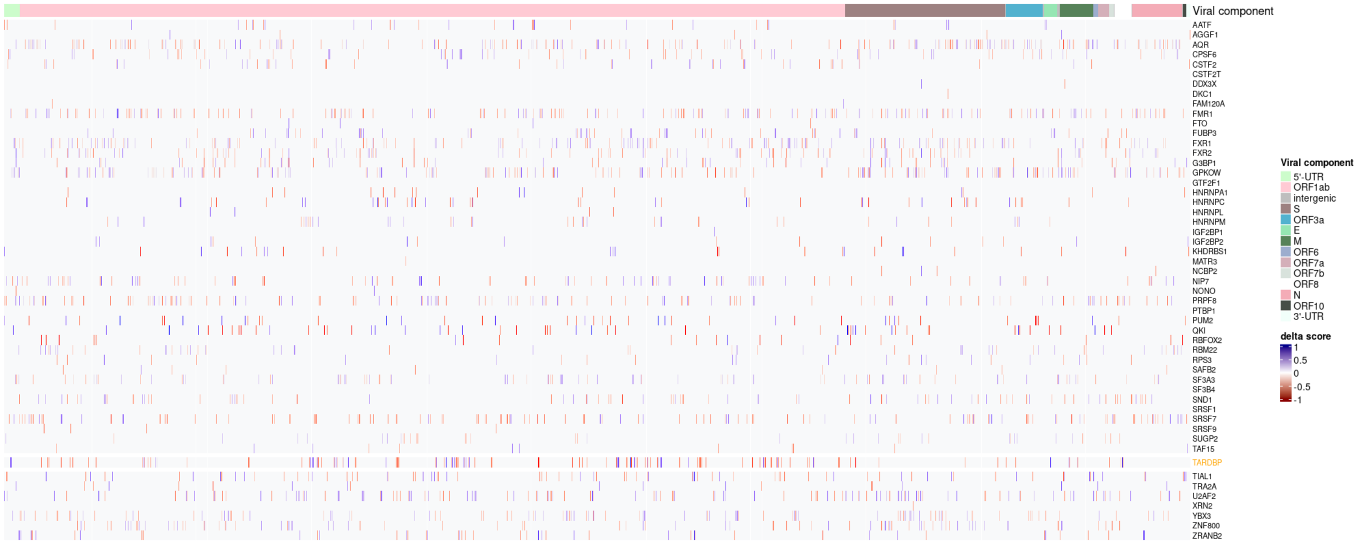

Supplementary Figure 4: In-silico perturbation analysis of SARS-CoV-2. Nucleotides across the viral genome were perturbed towards the three alternative bases and the alternative base with resulting the highest delta score considered for downstream analysis. Here, we show the delta-score heatmap across positions with at least one gain-or loss-of-binding event across all RBPs.

References

[1] Dae-Kyum Kim, Benjamin Weller, Chung-Wen Lin, Dayag Sheykhkarimli, Jennifer J. Knapp, Nishka Kishore, Mayra Sauer, Ashyad Rayhan, Veronika Young, Nora Marin-de la Rosa, Oxana Pogoutse, et al. A map of binary sars-cov-2 protein interactions implicates host immune regulation and ubiquitination. bioRxiv, 2021.

[2] David E. Gordon, Joseph Hiatt, Mehdi Bouhaddou, Veronica V. Rezelj, Svenja Ulferts, et al. Comparative host-coronavirus protein interaction networks reveal pan-viral disease mechanisms. Science, 370(6521):eabe9403, 2020.

[3] Jin Wei, Mia Madel Alfajaro, Peter C. DeWeirdt, Ruth E. Hanna, William J. Lu-Culligan, Wesley L. Cai, Madison S. Strine, Shang-Min Zhang, Vincent R. Graziano, Cameron O. Schmitz, et al. Genome-wide crispr screens reveal host factors critical for sars-cov-2 infection. Cell, 184(1):76–91.e13, 2021.

[4] William M. Schneider, Joseph M. Luna, H.-Heinrich Hoffmann, Francisco J. Sánchez-Rivera, Andrew A. Leal, Alison W. Ashbrook, Jérémie Le Pen, Inna Ricardo-Lax, Eleftherios Michailidis, Avery Peace, Ansgar F. Stenzel, Scott W. Lowe, Margaret R. MacDonald, Charles M. Rice, and John T. Poirier. Genome-scale identification of sars-cov-2 and pan-coronavirus host factor networks. Cell, 184(1):120–132.e14, 2021.

[5] Nora Schmidt, Caleb A Lareau, Hasmik Keshishian, Sabina Ganskih, Cornelius Schneider, Thomas Hennig, Randy Melanson, Simone Werner, Yuanjie Wei, Matthias Zimmer, et al. The sars-cov-2 rna–protein interactome in infected human cells. Nature Microbiology, 6(3):339–353, 2021.

[6] Ryan A Flynn, Julia A Belk, Yanyan Qi, Yuki Yasumoto, Jin Wei, Mia Madel Alfajaro, Quanming Shi, Maxwell R Mumbach, Aditi Limaye, Peter C DeWeirdt, et al. Discovery and functional interrogation of sars-cov-2 rna-host protein interactions. Cell, 184(9):2394–2411, 2021.

[7] Andrea Vandelli, Michele Monti, Edoardo Milanetti, Alexandros Armaos, Jakob Rupert, Elsa Zacco, Elias Bechara, Riccardo Delli Ponti, and Gian Gaetano Tartaglia. Structural analysis of sars-cov-2 genome and predictions of the human interactome. Nucleic Acids Research, 48(20):11270–11283, 2020.

[8] Denisa Bojkova, Kevin Klann, Benjamin Koch, Marek Widera, David Krause, Sandra Ciesek, Jindrich Cinatl, and Christian Münch. Proteomics of sars-cov-2-infected host cells reveals therapy targets. Nature, 583(7816):469–472, 2020.

[9] Sungyul Lee, Young-suk Lee, Yeon Choi, Ahyeon Son, Youngran Park, Kyung-Min Lee, Jeesoo Kim, Jong-Seo Kim, and V Narry Kim. The sars-cov-2 rna interactome. Molecular Cell, 2021.

[10] Alexey Stukalov, Virginie Girault, Vincent Grass, Ozge Karayel, Valter Bergant, Christian Urban, Darya A. Haas, Yiqi Huang, Lila Oubraham, Anqi Wang, M. Sabri Hamad, et al. Multilevel proteomics reveals host perturbations by sars-cov-2 and sars-cov. Nature, 594(7862):246–252, 2021.

[11] Lei Sun, Pan Li, Xiaohui Ju, Jian Rao, Wenze Huang, Lili Ren, Shaojun Zhang, Tuanlin Xiong, Kui Xu, Xiaolin Zhou, Mingli Gong, Eric Miska, Qiang Ding, Jianwei Wang, and Qiangfeng Cliff Zhang. In vivo structural characterization of the sars-cov-2 rna genome identifies host proteins vulnerable to repurposed drugs. Cell, 184(7):1865–1883.e20, 2021.
